# Katanin Localization Requires Triplet Microtubules in *Chlamydomonas reinhardtii*

**DOI:** 10.1371/journal.pone.0053940

**Published:** 2013-01-08

**Authors:** Jessica M. Esparza, Eileen O’Toole, Linya Li, Thomas H. Giddings, Benjamin Kozak, Alison J. Albee, Susan K. Dutcher

**Affiliations:** Department of Genetics, Washington University School of Medicine, St. Louis, Missouri, United States of America; University of Exeter, United Kingdom

## Abstract

Centrioles and basal bodies are essential for a variety of cellular processes that include the recruitment of proteins to these structures for both centrosomal and ciliary function. This recruitment is compromised when centriole/basal body assembly is defective. Mutations that cause basal body assembly defects confer supersensitivity to Taxol. These include *bld2*, *bld10, bld12*, *uni3, vfl1, vfl2*, and *vfl3.* Flagellar motility mutants do not confer sensitivity with the exception of mutations in the p60 (*pf19*) and p80 *(pf15)* subunits of the microtubule severing protein katanin. We have identified additional *pf15* and *bld2* (ε-tubulin) alleles in screens for Taxol sensitivity. Null *pf15* and *bld2* alleles are viable and are not essential genes in *Chlamydomonas*. Analysis of double mutant strains with the *pf15-3* and *bld2-6* null alleles suggests that basal bodies in *Chlamydomonas* may recruit additional proteins beyond katanin that affect spindle microtubule stability. The *bld2-5* allele is a hypomorphic allele and its phenotype is modulated by nutritional cues. Basal bodies in *bld2-5* cells are missing proximal ends. The basal body mutants show aberrant localization of an epitope-tagged p80 subunit of katanin. Unlike IFT proteins, katanin p80 does not localize to the transition fibers of the basal bodies based on an analysis of the *uni1* mutant as well as the lack of colocalization of katanin p80 with IFT74. We suggest that the triplet microtubules are likely to play a key role in katanin p80 recruitment to the basal body of *Chlamydomonas* rather than the transition fibers that are needed for IFT localization.

## Introduction

Taxol is a natural product that binds to β-tubulin and stabilizes microtubules in eukaryotic cells. Taxol is used for treatment of a variety of cancers as it blocks mitosis [1]. In the unicellular green alga, *Chlamydomonas reinhardtii,* Schibler and Huang showed that a mutation in β-tubulin (*tub2-1*) confers resistance to colchicine and supersensitivity to Taxol. They concluded that the microtubules in *tub2-1* cells are hyperstabilized, which causes the Taxol supersensitive phenotype [2]. Katanin is a microtubule severing protein [3], [4]. It is composed of two subunits; the p60 subunit is a catalytic AAA ATPase and the p80 subunit targets the heterodimer to the centrosome in metazoans. Katanin influences microtubule dynamics through its ability to sever microtubules. This property is observed in *Drosophila* S2 mitotic spindles [5] and *C. elegans* meiotic spindles [6]. Sharma and colleagues [7] showed that loss of either the catalytic (p60) or the targeting (p80) subunit of katanin in *Tetrahymena* results in short cilia and a knockout allele of p60 confers Taxol supersensitivity. Thus, there appear to be several pathways in cells that when mutated confer Taxol sensitivity.

Because katanin localizes to the centrosome, this localization seems likely to require intact centrioles/basal bodies. Centrioles are a component of the metazoan centrosome and help to recruit pericentriolar material (PCM) that nucleates both cytoplasmic and spindle microtubules [8], [9]. Functional centrioles and intact subdistal appendages are required for the recruitment of PCM proteins in animals. SPD-2, SPD-5 and SAS-4, which were first identified in *C*. *elegans* and localize to centrioles by immuno-electron microscopy, play essential roles in centriole biogenesis and they are needed to recruit γ-tubulin and aurora kinase to the PCM [9]–[11]. Sas-4 is thought to play a key role as it shows binding to αβ-tubulin dimers [12] as well as with Sas-5 and Cep152/Asl. Recruitment in *Drosophila* requires Asl (Cep152) and D-Spd2 (Cep192) [13]. The SPD-2/Cep192 homolog and centrosomin (Cnn), localizes to the centrioles and *spd2* mutants show significantly reduced concentrations of several centrosomal proteins that include Cnn, γ-tubulin, Dd4/Dgrip91, and D-TACC [8], [14]. Subdistal appendages on mature centrioles contain ninein [15]. Tissue culture cells depleted of ninein by siRNA show a significant reduction in γ-tubulin and the complete absence of the protein, astrin, at the centrosome [16]. Thus, defective centrioles or depletion of centriolar proteins prevent recruitment of some or all pericentriolar proteins.

When a centriole matures and converts to a basal body, it recruits intraflagellar transport (IFT) proteins and motors [17], [18]. Just as centriolar proteins are required to recruit PCM components for cytoplasmic and spindle microtubules, defective basal bodies disrupt localization of IFT proteins. In wild-type *Chlamydomonas* cells, IFT proteins accumulate around the basal body in a horseshoe-shaped structure [19], [20]. The *bld2-1* mutant has an incompletely assembled basal body [21], and although the IFT proteins are present, their localization is diffuse rather than in the horseshoe-shaped pattern.

We reasoned that *Chlamydomonas* mutants with basal body defects that lack the ability to dock intraflagellar transport proteins correctly could also fail to localize PCM components properly. We report a new phenotype that is associated with basal body biogenesis defects; these mutant strains show increased sensitivity to the microtubule-stabilizing drug, Taxol. We hypothesize that lack of localization or mislocalization of PCM-targeted proteins contributes to the Taxol supersensitivity phenotype.

Strains with basal body integrity defects fall into several classes. The first class shows defects in the assembly of the microtubule blades and includes *bld2*, *bld10*, *bld12*, *uni2* and *uni3*. The *bld2, bld10*, and *bld12* mutants lack complete microtubule blades, and *uni3* mutants lack triplet microtubules [22]–[25]. The *uni1* and *uni2* mutant shows a defect in the transition zone [26], [27] and the *uni1*; *uni2* double mutant affects the change from triplet to doublet microtubules [28]. The second class of mutants shows defects in the fibers that are required to maintain proper basal body orientation and segregation and include *vfl1*, *vfl2*, and *vfl3* [29]–[31].

We screened existing basal body and flagellar mutants for increased sensitivity to the microtubule-stabilizing drug, Taxol, as well as performed several screens for additional mutants that confer Taxol sensitivity. We find that katanin mutants in *Chlamydomonas* confer Taxol sensitivity and that mutants with basal body defects confer Taxol sensitivity, and have abnormal recruitment of the p80 katanin subunit to the basal bodies.

## Materials and Methods

### Cell Culture, Genetic and Phenotypic Analyses

*Chlamydomonas reinhardtii* growth conditions [32], matings [33], and revertant isolation using ultraviolet irradiation [34] were performed as previously described. Aflagellate strains were mated with 100 mM dibutyryl cyclic AMP (Sigma-Aldrich, St. Louis, MO) and 30 mM isobutyl 1-methylxanthine (Aldrich, Milwaukee, WI) [33]. For each sample, 200 cells were counted after fixation with 1% glutaraldehyde in phosphate buffer (pH 7.4). Taxol (paciltaxel, Sigma-Aldrich) was tested at concentrations varying from 2 µM to 18 µM in DMSO. Media with Taxol were kept in the dark for storage and in yellow Lucite boxes during growth to prevent its break down [35]. To determine if cells recovered from exposure to Taxol, cells were stained with 0.01% Trypan blue (Sigma-Aldrich). Oryzalin was a gift of Eli Lilly and Company. Oryzalin and colchicine (Sigma-Aldrich) were tested at 0.5–1 µM and 1–5 µM, respectively. The drugs, canavanine, cycloheximide, glyphosate, tunicamycin, methionine sulfoximine, anisomycin, erthyromycin, spectinomycin, 0.1% NP-40, 5-methyl anthranilic acid, and 3-aminopyridine were tested at a 2, 3, and 4 fold higher and lower than in [36]. Cleavage furrow placement measurements were performed as previously described [34], except ImageJ (NIH) was used to measure the area of the cells. Permutation tests were performed using 1000 permutations to determine significance [37].

The *tub2-1* (β_2_-tubulin mutant that was first published as *colR4, pf15-1*, *pf19-*1, *uni2-2, vfl1-1*, *vfl2-1* and *vfl3-1* strains were obtained from the *Chlamydomonas* Genetics Center. The *PF15* vector (pPF15) was obtained from E. Smith (Dartmouth University). The *bld10-1* and *bld12-1* strains were obtained from M. Hirono (University of Tokyo).

For synchrony, cells were grown in high salt medium [38] with a 14∶10 light: dark cycle as described previously [39] at 21°C and were maintained at a density of 1–3×10^5^ cells/ml. Samples were taken at 15 and 30 min time points beginning at 15 min into the dark phase.

### Mutagenesis and Isolation of new *bld2* Alleles

To isolate new alleles by noncomplementation, *bld2-2 NIT2 ac17/BLD2 nit2-1 AC17* diploid cells were used as previously described [34]. Diploid cells were grown on rich (R) solid medium and subjected to 75,000 µJoules/cm^2^ of ultraviolet irradiation and allowed to recover in the dark for 24 hours. Each plate was divided into 12 sections and each section was placed individually into 20 ml R liquid medium in 25×150 mm culture tubes. Cells at the bottom of the tube were transferred to new tubes containing 20 ml R medium every 2–3 days for a total of 12 times and 10 µl from the bottom of the last tube was plated onto solid R medium. Individual colonies were picked into 2.5 ml R liquid medium and assayed for their ability to oppose gravity.

To differentiate between mutations resulting from chromosome loss or mitotic recombination, dominant enhancers, and new alleles we took advantage of the absence of a *Fok*I restriction enzyme site in the *bld2-2* allele. PCR amplification of the region around the mutation and digestion with the *Fok*I restriction enzyme produces both the digested wild-type product and uncut *bld2-2* product in the heterozygous parental diploid. If mitotic recombination or chromosome loss occurs, only the *bld2-2* fragment would be amplified by PCR [34].

To isolate new alleles in haploid strains, an insertional mutant collection with 3000 independent strains, which was a kind gift from Dr. Lauren Mets (University of Chicago), was used. It was constructed in the CC-125 strain by transformation with the *ble* gene [40]. The collection was screened by replica plating using RepliPlate pads (FMC, Rockland, ME) onto solid R medium with 8 µM Taxol at 25°C. Plates containing Taxol were maintained in yellow Lucite boxes [35].

### PCR and Sequencing

Genomic DNA from *bld2-5* and *bld2-6* cells was isolated using a modified protocol of the Genisol Maxi-Prep Kit (Abgene, Rockford, IL). Between 10^5^–10^6^ cells were suspended in 50 µl of 1X Tris-EDTA buffer with a 100-fold reduction in the suggested volumes. Primers for sequencing were described previously and are available upon request. REDTaq DNA polymerase (Sigma-Aldrich) was used with the following conditions: 31 cycles of 1 min at 95°C, 1 min at 57°C and 1 min at 72°C followed by a 10 min extension at 72°C. PCR products were column purified (QIAGEN, Valencia, CA) and then cycle sequenced using the following conditions: 2 min at 96°C and 32 cycles of 96°C for 10 sec, 50°C for 5 sec and 60°C for 4 min. Sequencing reactions were precipitated with the addition of 125 mM EDTA and 100% ethanol and incubated for 15 min at room temperature (RT). Reactions were centrifuged, washed with 70% ethanol and dried before the addition of Hi-Di formamide (Applied Biosystems, Foster City, CA). After 2 min incubation at 95°C, the reactions were loaded onto a 3100 Genetic Analyzer (Applied Biosystems). Sequenced data were aligned and analyzed with Sequencher (Gene Codes, Ann Arbor, MI). Genomic DNA from the eight intragenic revertants was isolated and sequenced as described above with primers that produce a 367 bp fragment (tns-28-1f; (GTGACAACGGGGAACTAAGC and tns-28-1r:GACAGCTGCTGCATTGTGAT). TAIL PCR determined the insertion site of the *ble* gene in the *bld2-6* allele [41], [42] using primers in Table S2.

### Construction of *PF15* Transgene by Knitting PCR

The pPF15 vector, provided by Elizabeth Smith (Dartmouth University), was used to amplify two fragments of 304 and 195 bp for knitting PCR [43] using the primers PF15 FRAG F/PF15 NOT R (CCCTCCTCGCCCAGGTGATG , CTAGCGGCCGCGCTGCGCCAGCTG ) and PF15 NOT F/PF15 FRAG R (CAGCTGGCGCAG CGCGGCCGCTAG, CATTCGTCCTGCAGGGCCAC). The PF15 NOT primers contain the *Not*I restriction enzyme site and it changes the last amino acid of *PF15* from a leucine to an asparagine. These fragments were amplified using Klentaq Long and Accurate polymerase using the following conditions: 30 cycles of 1 min at 95°C, 1 min at 56°C and 1 min at 68°C, followed by a 30 min extension at 68°C. The fragments were purified from a 2% agarose gel (Gel Purification Kit; MO BIO Laboratories Inc., Carlsbad, CA). The above PCR was repeated using equal quantities of each PCR product and the PF15 FRAG primers to incorporate a *Not*I restriction site that was used to clone the HA tag into the gene (Figure S1). The resulting approximately 500 bp fragment was gel purified and cloned into the pCR4-TOPO vector (Invitrogen, Carlsbad, CA). Transformed colonies were verified by colony PCR and used to isolate plasmid DNA with the Wizard Plus SV Minipreps DNA Purification System (Promega, Madison, WI). The PF15 fragment with the incorporated *Not*I site (pPF15-N) was digested with *Eco*RI, gel purified as described above, and ligated to the LITMUS 28i vector (New England Biolabs, Ipswich, MA). Transformed colonies were verified by colony PCR using the PF15 FRAG primers. The HA tag was ligated separately into pPF15-N plasmids. Positive colonies were assayed for number of tags and orientation by PCR and digestion. Multiple tagged *PF15* genes were transformed into the *pf15* mutant strain by electroporation [44], [45] and transformants were screened by their ability to oppose gravity.

### Preparation of Cells for Electron Microscopy

Cells were prepared for electron microscopy using methods described in O’Toole *et al*. [46], [47]. Briefly, aliquots of cells grown in suspension were spun at 500×g and then resuspended in 150 mM mannitol. The samples were spun again at 500×g and the resulting loose cell pellet was then transferred to aluminum sample holders and rapidly frozen in a Balzers HPM010 high pressure freezer (BAL-TEC, Technotrade International, Manchester, NH). The frozen cells were freeze-substituted for three days at −90°C in 1% OsO_4_ and 0.1% uranyl acetate in acetone, warmed to room temperature and embedded in epon/araldite resin.

Serial thin (50–70 nm) or thick (250–400 nm) sections were cut using an Ultracut-E microtome (Leica, Germany) and the section ribbons were collected onto Formvar-coated copper slot grids. The sections were post-stained in 2% uranyl acetate in 70% methanol followed by Reynold’s lead citrate. For tomography, 15 nm colloidal gold particles were used (Sigma-Aldrich).

### Electron Microscopy

Serial thin sections were imaged in a Philips CM10 EM (FEI, Mahwah, NJ) operating at 80 kV. Serial sections of the basal body through the transition zone from 13 cells were collected to document the phenotype and aid in the interpretation of tomographic data.

Electron tomography was carried out essentially as described [47], [48]. The specimens were placed in a tilt-rotate specimen holder (Gatan, Pleasanton, CA) and tomographic data sets recorded using a TECNAI F30 intermediate-voltage electron microscope (FEI, The Netherlands) operated at 300 kV. Images were captured every 1° over a ±60° range using a Gatan 2 K×2 K CCD camera at a pixel size of 1 nm. The grid was rotated 90°, and a second tilt series was acquired. Dual-axis tomographic reconstruction was carried out using the IMOD software package as previously described [46], [49], [50]. Briefly, the tilted views were aligned using the positions of the colloidal gold particles, and tomograms were calculated using an R-weighted back projection algorithm. The two tomograms were then aligned to each other and combined. Finally, dual-axis tomograms from serial sections were aligned and combined using the methods described by O’Toole *et al*. [46]. A total of 7 dual-axis tomograms were reconstructed to examine the 3-D fine structure of the *bld2-5* basal bodies.

### Indirect Immunofluorescence

Interphase cells were treated with autolysin to remove cell walls [51], [52] and resuspended in MT buffer [53] and 12% hexylene glycol [32] and applied to slides pretreated with poly-L-lysine (Sigma-Aldrich) for 5 min at RT and dried. Slides were incubated in methanol prechilled to −20°C for 10 min. Slides were rehydrated in 1× PBS and incubated in blocking solution (12.5% BSA, 0.01% cold water fish gelatin (Sigma-Aldrich)) in 1× PBS for 30 min. Slides were transferred to blocking solution with 10% newborn goat serum (Accurate Chemical, Westbury, NY) and incubated for 30 min at RT without agitation. Primary antibodies were diluted in blocking solution and incubated overnight at 4°C with the following dilutions; anti-acetylated α-tubulin (1∶1000, Sigma-Aldrich), anti-centrin (1∶1000; kindly provided by Dr. Jeff Salisbury, Mayo Clinic), anti-HA (500 ng/µl; Roche, Indianapolis, In), anti-IFT74 (1∶600; kindly provided by Dr. Carlo Iomini), and anti-γ-tubulin (1∶1000; Sigma-Aldrich). Slides were washed in 10% blocking solution three times with agitation, 10 min each, at RT and incubated in secondary antibody for 1 hr at RT. Alexa 594 and Alexa 488 mouse and rabbit secondary antibody (Invitrogen) were used at 1∶1000 dilution in blocking solution. Slides were washed three times with agitation, 10 min each, at RT and mounted with Vectashield (Vector Laboratories, Burlingame, CA). Images were collected on an Axiophot microscope modified with a Lambda DG-4 light source (Sutter Instrument Company, Novato, CA) equipped with a Photometrics Cascade 512B camera (Roper Scientific, Tucson, AZ) and a Physick Instrument piezoelectric stage (Karlsruhe, Germany). Slidebook Digital Software was used for deconvolution of the images (Intelligent Imaging Innovations, Denver, CO) on a Dell dual processor computer (Round Rock, TX). Images were exported to Adobe Photoshop CS2 (Adobe Systems, Mountain View, CA).

Images with synchronized cells were obtained with a PerkinElmer UltraVIEW VoX laser scanning disk confocal system equipped with a Zeiss AxioObserver Z1 microscope, a-Plan-Apochromat 100×/1.46 oil DIC M27 objective, and EMCCD camera. Images were acquired with Volocity software and are displayed as maximum projections assembled from a z-stack. Pictures were assembled in Adobe Illustrator.

### Immunoblotting

Protein extracts from intact cells were prepared from equal numbers of cells of each strain and mixed with Laemmli sample buffer (Biorad, Hercules, CA) and 2% 2-mercaptoethanol. The samples were boiled for 5 min and centrifuged for 1 min to pellet cell debris before loading supernatant onto the gel. Proteins from intact cells were size-fractionated on SDS-PAGE minigels (1.0 mM thick, 10% acrylamide; 29∶1 with Bis-acrylamide) and transferred to Immobilon-P membranes (Millipore; Billerica, MA) in 20% methanol at 50 V for 1 hour. The rat anti-HA high-affinity antibody (200 ng/ml) and chicken anti-PbsA (1∶10,000, Agrisera; Sweden) were diluted in 5% milk in PBS. Secondary antibodies, donkey anti-rat HRP (1∶10,000, Jackson Labs) and rabbit anti-chicken IgY, HRP (1;10,000, Promega) were diluted in 5% milk in PBS. Lumi-Light Western blotting substrate (Roche) was used for detection and exposure to Super RX x-ray film (Fujifilm, Stamford, CT). Using Image J, the expression of katanin p80 was normalized to the PbsA control by measuring the pixels within a constant area and calculating a ratio for each to be compared to *pf15; PF15HA*.

## Results

### Screening Existing *Chlamydomonas* Mutants for Taxol Supersensitivity

**Figure 1:**
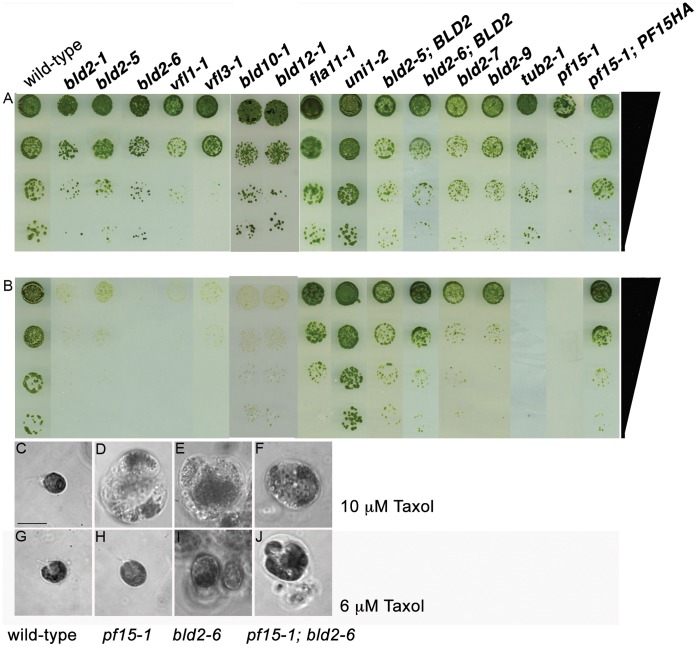
Basal body mutant strains show supersensitivity to Taxol. (A) Serial dilution of mutant, rescued, and intragenic revertant strains on control medium and (B) 8 µM Taxol-containing medium. Phase images of cells on media with different Taxol concentrations. (C, G) Wild-type, (D, H) *pf15-1*, (E, I) *bld2-6* and (F, J) *bld2-6, pf15-1* double mutant on 10 µM (C–F) or 6 µM Taxol (G–J) containing medium. The *bld2-6, pf15-1* double mutant is unable to grow on 6 µM Taxol containing medium compared to the single mutant strains. Scale bar in Panel C equals 10 µm. Panels C–J are at the same magnification.

Missense mutations in *pf15* mutant, which encodes the katanin p80 subunit and *pf19-1*, which encodes the katanin p60 subunit, were originally identified as paralyzed flagellar mutants with a defect in the central pair microtubules [54] [55], [56]. Similar to the loss of the p60 katanin subunit in *Tetrahymena* [7], these mutants confer sensitivity to Taxol (Figure 1). Wild-type cells arrest as swollen cells on 18 µM Taxol medium, while *pf15-1* and *pf19-1* mutant cells become swollen and do not divide on 8 to 18 µM Taxol media (Figure 1C, G). We screened other mutants with defects in axonemal substructures needed for motility (*pf2*, *pf9*, *pf14*, *pf16*, *pf17*, *pf18*, *oda2, ida3*) [55], [57], [58]and only the two katanin mutants confer Taxol supersensitivity.

We next examined the role of basal bodies in this phenotype. The *bld2-1* [34], *uni3-1* [22], *bld10-1* [23], *bld12-1* [25], *vfl1* [30], *vfl2-1* [31], and *vfl3* [29] mutations confer Taxol supersensitivity (Figure 1A, B), while the *uni1-2* [26] and the *uni2-2* [27] mutants do not confer supersensitivity. Since a majority of these strains have a high proportion of aflagellate cells, we asked if the Taxol phenotype was related to the aflagellate phenotype or the basal body phenotype. Mutants that lack flagella due to defects in IFT proteins and motors (*bld1*, *ift80* at 21°C and *fla8*, *fla10*, *fla11*, *fla15*, *fla17* at 32°C) [20], [59]–[65] do not show Taxol supersensitivity (Figure 1A, B). To ask if the Taxol supersensitivity reflects a general defect in drug uptake or export, we examined the *bld2-1* and *pf15-1* strains for resistance or sensitivity to other inhibitors (see Materials and Methods for a list). No resistance or supersensitivity was observed, which suggests that the Taxol supersensitivity of the *bld2* and *pf15* alleles does not result from defective import or export. In summary, multiple basal body mutants and the katanin mutants confer Taxol sensitivity. The wild-type strains show similar swelling but require increased concentrations of Taxol (Figure 1 C–J).

It is likely that multiple proteins may require intact basal bodies for localization and some of these may influence microtubule dynamics. Thus, the Taxol phenotype of the basal body mutants could arise from a failure to recruit katanin or a failure to recruit katanin and other proteins. This can be tested in double mutants of null mutants of katanin and basal body proteins. Therefore, we sought to identify null alleles of *pf15* and *bld2.*

### Isolation of *pf15-3* as a Taxol Supersensitive Mutant

Wild-type cells were mutagenized with ultraviolet light and 100 independent, nonmotile strains were isolated and 12 of these confer Taxol supersensitivity. One of these strains had immotile flagella. Three lines of evidence show that it carries a new *pf15* allele. The strain was mapped and is tightly linked to the *PF15* locus in 36 tetrads. The gene was sequenced and a C to T change was observed that generates a nonsense codon at amino acid Q_59_. The immotile cilia defect and the Taxol supersensitivity is rescued by the *PF15::HA* transgene (described below) in 17 independent transformants. The rescued strains have flagella with normal length and motility. The new allele is likely to be a null allele and the phenotype of the new *pf15-3* strain has similar phenotypes to the original *pf15-1* allele, although the presence of central pair microtubules has not been determined in the *pf15-3* mutant.

### Isolation of a Taxol Supersensitive *bld2-6* Allele

A collection of 3000 mutant strains made by insertional mutagenesis with the *ble* gene, which confers Zeocin resistance, [40] was screened for the failure to form colonies on 8 µM Taxol medium. Thirty-five strains showed Taxol supersensitivity and were crossed with a wild-type strain (CC-124) to determine if the insertional *ble* marker cosegregates with the Taxol phenotype. Cosegregation of Zeocin resistance and Taxol supersensitivity was observed in only one strain (strain ble12), which suggests that the selection on Zeocin medium was highly mutagenic as this high frequency of a lack of cosegregation is not observed in other insertional collections. In the ble12 strain, the Taxol phenotype (Figure 1A, B) and resistance to Zeocin cosegregates in 370 tetrads. This strain has a third phenotype; it completely lacks flagella (Table 1) and this phenotype cosegregates as well. The three phenotypes map to linkage group III near the *NIT2* locus based on crosses to CC-1952. The ble12 strain fails to complement the *bld2-2* and *bld2-5* mutations (see below) for the Taxol and flagellar phenotypes in 8 independent diploid strains for each allele. It also fails to complement the *bld2-2* mutation for the meiotic phenotype; no viable progeny were recovered from 104 tetrads of this cross. PCR with 21 primer pairs in this region revealed that ble12 carries a 12.6 kb deletion that removes the ε-tubulin gene and the coding region of the *PRMT1* gene, which encodes a protein methyltransferase protein (Table S2). The ble12 mutant allele is named *bld2-6*.

**Table 1:** Numbers of flagella in *bld2-5*, *bld2-6* and intragenic revertant strains.

		% cells with flagella numbers of			
Strain	Temperature(°C)	0	1	2	>3
**Logarithmic**					
*BLD2*	25	5	3.5	91.5	0
*BLD2*	32	16	7	77	0
*bld2-1*	25	100	0	0	0
*bld2-1; BLD2TG*	25	5.5	7.5	87	0
*bld2-6*	25	100	0	0	0
*bld2-6*; *BLD2TG*	25	22.5	3.5	72.5	1.5
*bld2-5*	14	100	0	0	0
*bld2-5*	21	100	0	0	0
*bld2-5*	25	95	4	1	0
*bld2-5*	32	95	4.5	0	0.5
*bld2-5; BLD2TG* a	25	6.3	14.8	80.3	0
*bld2-7*	25	2.5	4	93.5	0
*bld2-9*	25	7	3	90	0
*BLD2/BLD2*	25	15	9	76	0
*bld2-5/BLD2*	25	10.3	3.9	84.5	0.81
**Gametic**					
*bld2-5*	25	79	14	6.5	0
*bld2-5*	32	74	15.5	10.5	0
*bld2-6*	25	100	0	0	0
*bld2-7*	25	10	18.5	71.5	0
*bld2-9*	25	8.5	12	79.5	0

a *BLD2TG* indicates the ε-tubulin transgene described previously [24].

### *bld2; pf15* Double Mutants have an Additive Phenotype

The Taxol phenotype of the *bld2* alleles and the *pf15* strains is similar and both are unable to grow on 8 µM Taxol containing medium. Double mutants were constructed to ask if the phenotype of double mutants is more severe than either mutant alone. If there are other proteins that must be recruited, then an additive or synthetic phenotype should be observed using the two null alleles. Wild-type, *pf15-3*, *bld2-6, pf15-3*; bld2-6, *bld2-6; pf15-1,* and *bld2-6; pf15-3* were grown on 0, 4, 6, 10 µM Taxol containing medium. All strains grow on 4 µM and only the *bld2-6*, *pf15-1* and the *bld2-6; pf15-3* double mutant strains fail to grow on 6 µM Taxol containing medium while the single mutant strains form colonies on this concentration (Figure 1K). This additive phenotype suggests that the recruitment of other proteins besides katanin p80 may affect microtubule dynamics. This is not surprising in that over 100 proteins have postulated to reside at the centrosome [66].

### Characterization of the *bld2-4* allele

We had previously reported that ε-tubulin is an essential gene based on the *bld2-4* allele [34]. The *bld2-4* allele is an insertional mutation that has a dominant lethal meiotic phenotype. Genetic analysis showed that the *bld2-4* allele could only be recovered in a disomic background, which suggested that ε-tubulin was an essential gene in *Chlamydomonas*. The isolation of the *bld2-6* brought into question if ε-tubulin is an essential gene. To ask if the *BLD2* transgene is sufficient to rescue the lethal phenotype [34], we used the disomic *bld2-1; bld2-4* in a series of crosses (Figure S2). We found that two copies of the *BLD2* gene are necessary to rescue the meiotic lethal phenotype and that the *BLD2* transgene fails to rescue the mitotic lethality. To ask if the insertion is associated with a deletion and to determine the extent of the deletion, progeny obtained from a cross with the polymorphic strain, CC-1952, were used. Progeny were scored by the presence of the *NIT2* allele from the CC-1952 parent, the absence of the *bld2-1* allele, the presence of the *BLD2* transgene. The extent of the deletion was estimated by the presence/absence of heterozygosity of physical markers surrounding the *BLD2* gene (Table S1). The deletion extends from position 4125635 on chromosome 3 to between 4026909 and 4021900, which removes 28 predicted genes. The dominant meiotic lethality is likely to be due to the loss of multiple genes given that the transgene rescues the meiotic phenotype of *bld2-6* (see below). Rescue of the mitotic lethality of *bld2-4* is likely to require additional flanking DNA and thus ε-tubulin is not an essential gene as reported previously [34].

### Isolation and Identification of the *bld2-5* Allele

Concurrently, we identified an additional *bld2* allele using a noncomplementation screen. A screen of phenotypically wild-type heterozygous *BLD2/bld2* diploid strains produced six strains that failed to swim. Non-complementation screens produce several outcomes in addition to new alleles, which include mitotic recombination, chromosome loss and unlinked dominant enhancers [34]. Two of the mutant strains resulted from mitotic recombination or chromosome loss based on the loss of the *Fok*I restriction site (see Material and Methods). The remaining four strains remained heterozygous at the *BLD2* locus and were mated to a *bld2* strain to determine if the new mutations were new alleles or unlinked dominant enhancers. Two of the mutants produced swimming progeny that suggest an unlinked dominant enhancer. The other two strains did not produce swimming progeny, which suggests new alleles. One of these strains (4-1) was characterized.

The 4-1 mutant strain was backcrossed twice to wild-type cells to remove unlinked mutations and to restore euploidy as judged by greater than 86% meiotic viability. The backcrossed 4-1 strain failed to oppose to gravity and lacked flagella and the aflagellate phenotype was used for mapping. The mutation failed to recombine with the *bld2-1* allele in 210 complete tetrads. We analyzed 1571 progeny from the cross of 4-1 to the polymorphic strain, CC-1952 [67] using dCAPs markers (Table S2). The 4-1 mutation maps to a 54.1 kb region that includes the *BLD2* gene [68] and gives a value of 102 kb per map unit for this region.

We sequenced ε-tubulin from the 4-1 strain and found a T to A transition that changes an isoleucine to an asparagine at amino acid 163 (I_163_N). Introduction of a wild-type copy of *BLD2* into the 4-1 strain through a cross with a *bld2-1* strain with an unlinked *BLD2* transgene (*BLD2 TG*) produced tetrads with two aflagellate progeny and two swimming progeny (n = 57). The *Fok*I restriction enzyme digest assay (see Materials and Methods) differentiates between swimming progeny with the *BLD2* transgene and the *bld2-1* allele (heterozygous) or the 4-1 allele (homozygous). Approximately one-half of the swimming progeny show the homozygous digestion pattern predicted for the 4-1 strain and the remainder shows the heterozygous pattern of wild-type and *bld2-1* (n = 6; data not shown). Thus, the transgene rescues the flagellar phenotype of the 4-1 allele to the same extent as it rescues the *bld2-1* allele (see Table 1). Unlike the other *bld2* alleles, the 4-1 allele does not display a meiotic phenotype in four homoallelic or 16 heteroallelic meiotic crosses of independent meiotic progeny.

The isoleucine is not highly conserved in ε-tubulin from a diverse range of organisms (7 of 28), therefore we screened for reversion of the aflagellate phenotype to provide further evidence that this change was responsible for the phenotypes. Cells were mutagenized with ultraviolet light and 25 independent strains that swim were isolated. In crosses of these swimming strains to wild-type cells, the aflagellate phenotype was not recovered in 8 of the 25 strains in at least 10 tetrads; these are likely to be intragenic revertants or tightly linked suppressors. The other 17 strains segregated the aflagellate phenotype, which indicates that they contain extragenic suppressor mutations. The characterization of these extragenic suppressors will be reported elsewhere. To determine if the mutation in the 4-1 strain is changed in the revertants, a 367 bp fragment containing the I_163_N mutation was sequenced. Four of the strains convert the asparagine back to isoleucine; these are true revertants. The remaining four strains are pseudorevertants. Two strains (T42 and T16) change the asparagine to serine (N_163_S) and the other two strains (T33 and T29) convert the serine at position 144 to a glycine (S_144_G, I_163_N), but retain the asparagine. The amino acids from ε-tubulin are compared to the corresponding amino acids of the β-tubulin crystal structure using Modeller [69]. The isoleucine of ε-tubulin lies in a hydrophobic region and the S_144_ lies in loop between a β-strand (B4) and a α-helix (H3), which is a part of the nucleotide binding region [70]. The change of a hydrophobic isoleucine to a hydrophilic asparagine should greatly change the properties of this region. In the T42 and T16 strains, asparagine is mutated to the smaller and more neutral amino acid serine. The identification of a mutation in the coding region of ε-tubulin, the rescue of the phenotype with a wild-type ε-tubulin transgene and the identification of both true and pseudorevertants suggest that the 4-1 strain is a new ε-tubulin allele that we call *bld2-5.* Furthermore, the pseudorevertants identify new *bld2* alleles: the T42 and T16 pseudorevertants are named *bld2-7* and *bld2-8* and the T33 and T29 pseudorevertants are named *bld2-9* and *bld2-10*. The *bld2-5* strain with an extra copy of *BLD2* (ε-tubulin) as well the *bld2-7* and *bld2-9* intragenic revertants do not show the Taxol supersensitivity phenotype (Figure 1A, B).

### Phenotypic Analysis of the *bld2-5* and Pseudorevertant Strains

The *bld2-5* strain exhibits a less severe flagellar assembly defect than observed in previously described *bld2* alleles. Ninety-five percent of the *bld2-5* cells are aflagellate, but 4% of the cells have one flagellum and 1% has two flagella in logarithmically grown cells (n = 200) (Table 1). Surprisingly, when *bld2-5* cells are deprived of nitrogen and arrested in G1 of the cell cycle as gametic cells, 20.5% of the cells assemble at least one flagellum. Changing the cell cycle time by growing cells at 14°C or 32°C does not change the number of flagellated cells (Table 1), which suggests the *bld2-5* allele is not temperature-sensitive, but may be modulated by nutritional cues. This is the only *bld2* allele that shows this phenotype.

**Figure 2:**
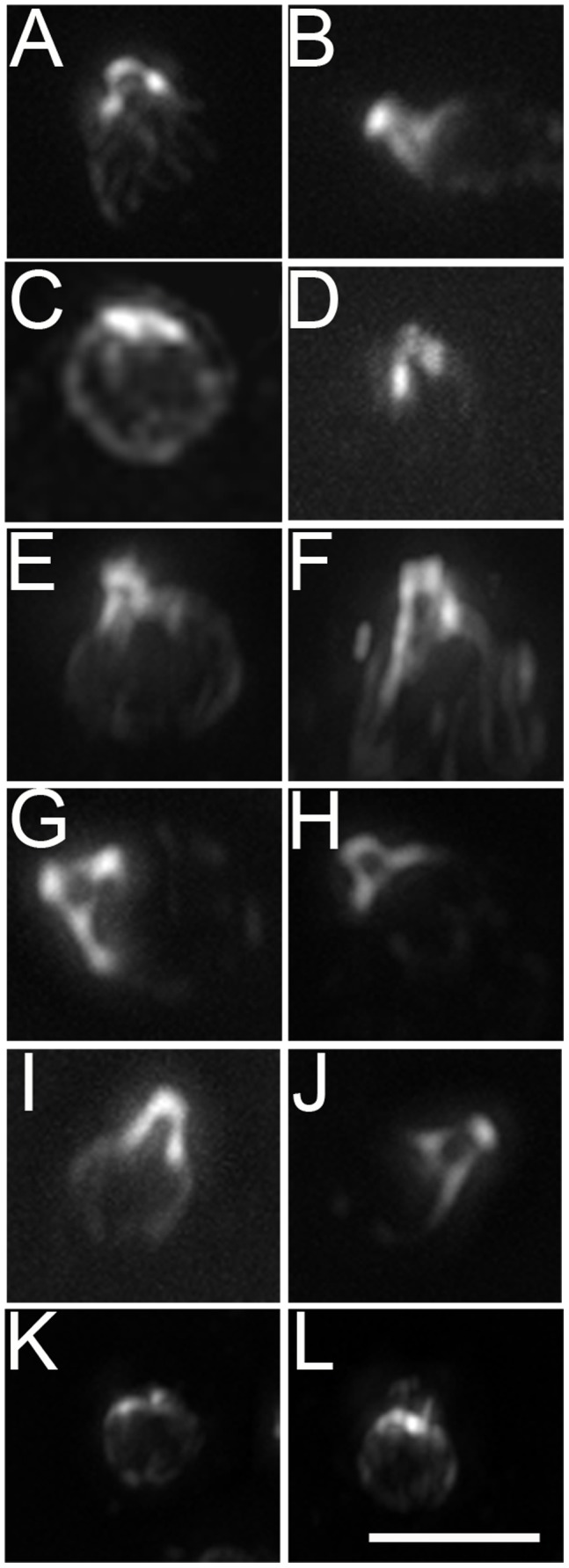
Centrin localization varies in the *bld2-5* and *bld2-6* strains. (A, B) Wild-type cells with an extended pattern of centrin. (C) Approximately 41% of *bld2-5* cells have centrin that collapses on the nucleus (n = 70). (D) *bld2-5* cells with a wild-type centrin pattern. (E, F) The rescued transformant, *bld2-5*; *BLD2*, and (G, H) the pseudorevertants, *bld2-7* and (I, J) *bld2-9* have extended centrin. (K, L) All *bld2-6 cells* show centrin collapses on or around the nucleus. Scale bar in panel L equals 10 µm. Panels A–L are at the same magnification.

In wild-type strains, centrin is a component of the distal striated fiber that connects the distal ends of the basal bodies, the stellate fibers of the transition zone, and extends as fibers from the basal body to the nucleus (the nucleo-basal body connector) [71] (Figure 2A, B). In *bld2-1* strains, centrin collapses on or around the nucleus [34], [72]. Collapsed centrin occurs in 41% of *bld2-5* cells (n = 50, Figure 2C) while the remainder has a wild-type localization pattern (Figure 2D). It appears that the presence of an extended nucleo-basal body connector does not guarantee flagellar assembly.

**Figure 3:**
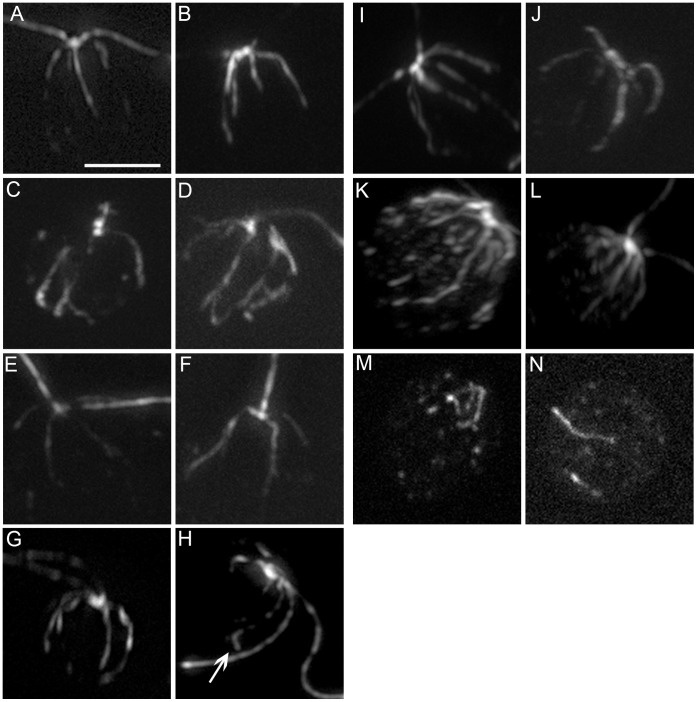
Rootlet microtubules are disorganized in the *bld2-5* and *bld2-6* strains. (A, B) Rootlet microtubules in wild-type cells form a cruicate pattern. (C, D) *bld2-5* cells show an aberrant number and placement of rootlet microtubules. (E, F) The *bld2-5*; *BLD2* strain shows a wild-type rootlet microtubule phenotype (N = 15). Pseudorevertants *bld2-7* (G, H) and *bld2-9* (I, J) have a nearly wild-type rootlet microtubule phenotype but splaying occurs at the ends of the microtubules (arrow). (K, L) The *tub2-1* strain has increased acetylated α-tubulin staining. (M, N) The *bld2-6* cells have a severe disorganization of rootlet microtubules. Scale bar in Panel A equals 5 µm. Panels A–N are at the same magnification.

**Figure 4:**
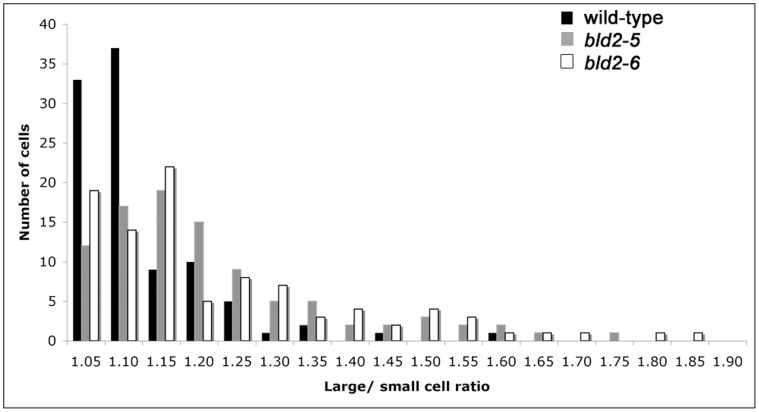
The *bld2-5* and *bld2-6* strains misplace the cleavage furrow. A. The ratio of the areas of wild-type sister cells is approximately equal to one (black bars), whereas the ratio of the areas of *bld2-5* (gray bars) and *bld2-6* (white bars) sister cells is equal to or greater than one, which suggests a defect in proper placement of the cleavage furrow [34]. These results are statistically significant compared by a permutation test [37].

In wild-type strains, acetylated α-tubulin labeling of rootlet microtubules forms a cruicate pattern [32], [73] Figure 3A, B). The *bld2-5* strain, like other *bld2* strains, shows disorganized rootlet microtubules in 95% of the cells (Figure 3C, D). The 5% of cells with wild-type rootlet microtubules are likely to have intact microtubule blades at the proximal end of the basal bodies [74]. Proper placement of the cleavage furrow depends on both centrin and rootlet microtubules [75]. Defects in these cytoskeletal structures cause aberrant cleavage furrow placement [76]. The area of newly divided daughter cells was measured to determine if the cleavage furrows were properly placed, since wild-type cells produce daughters with equal sizes [34]. Based on measurements of 100 pairs of cells, the *bld2-5* strain produces daughters with significantly different areas (p = 0.001), which indicates a defect in cleavage furrow placement (Figure 4A). The sum of *bld2-5* sister cells’ areas is significantly smaller than wild-type (p = 5×10^−14^, Figure 4B), which may suggest that the cells divide earlier than in wild-type cells. Both rescued transformants (*bld2-5*; *BLD2TG*) and intragenic revertants (*bld2-7* and *bld2-9*) assemble comparable numbers of flagella as wild-type cells (Table 1). They display wild-type centrin localization (Figure 2E–J). The transgene containing strain has wild-type rootlet microtubules (Figure 3E–J), but approximately 50% of rootlet microtubule bundles (n = 15) in the intragenic revertants appear to be slightly splayed at their ends (Figure 3H).

The *bld2-6* allele also shows centrin collapsed on the nucleus (Figure 2K, L) and disorganized rootlet microtubules (Figure 3M, N). *bld2-6* cells are 100% aflagellate as vegetative or gametic cells (Table 1). A single copy of the ε-tubulin gene rescues the flagellar, Taxol, and meiotic defects as well as centrin localization (n = 20) and the rootlet microtubules phenotypes of the *bld2-6* allele (n = 15).

### *bld2-5* Cells have Staggered Microtubule Blades Lengths in Mature Basal Bodies

**Figure 5:**
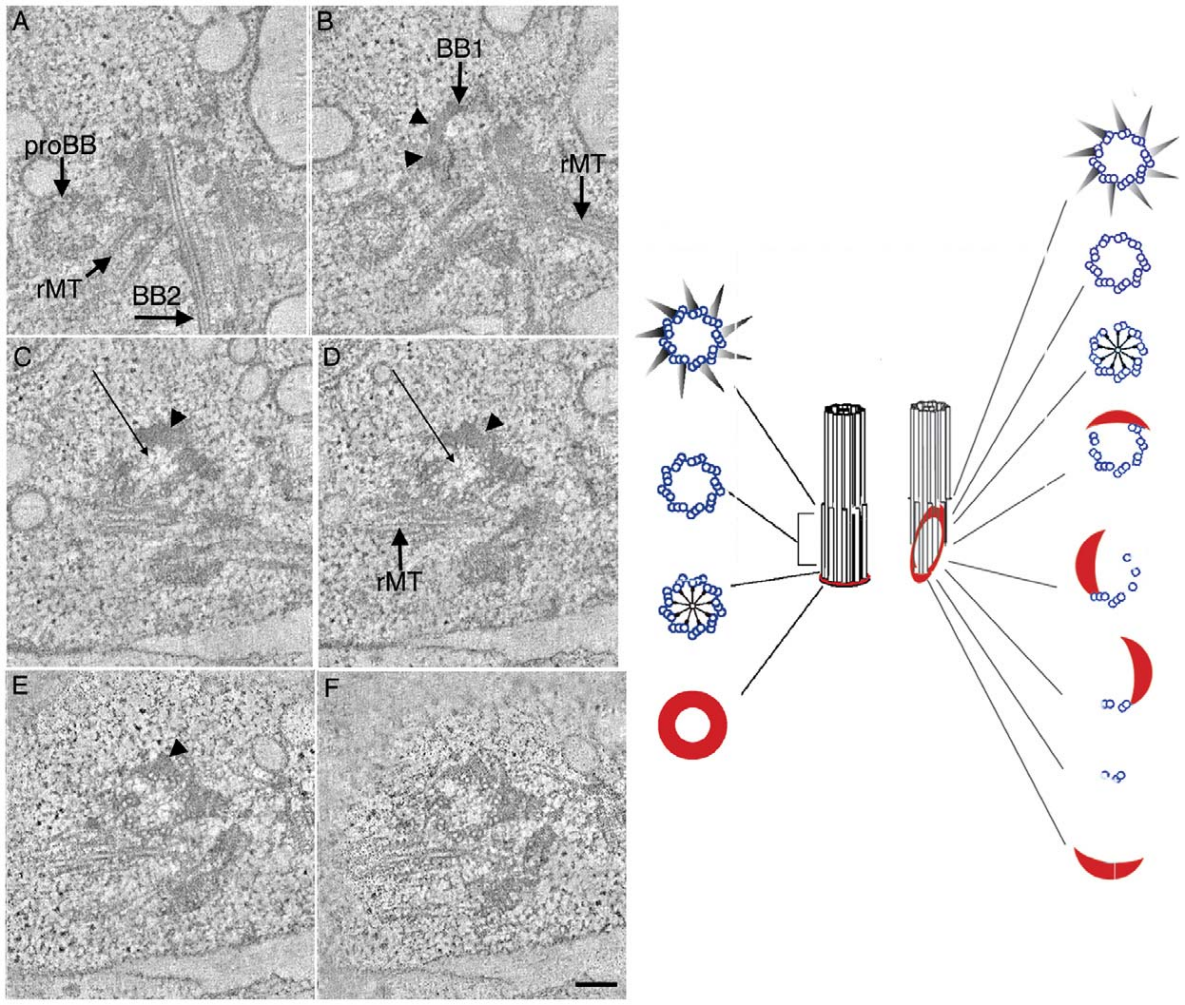
Mature basal bodies in the *bld2-5* strain contain defects in microtubule blade organization. Selected tomographic slices show the progression from the proximal (A) to the distal end (F) of the basal body. (A) Probasal bodies contain a ring of amorphous material at their proximal base. (B–E; arrowheads) Mature basal bodies (BB1) contain amorphous material that extends distally rather than in a proximal ring. (D–F) The assembly of microtubule blades is also incomplete with singlet, doublet and sometimes triplet microtubules present. (C, D; arrows) The cartwheel structure is observed distally. BB1, mature basal body 1; BB2, mature basal body 2; rMT, rootlet microtubules. Scale bar equals 100 nm and Panels A–F are at the same magnification. Schematic representation of the structure of a wild-type basal body and the defects in *bld2-5* basal bodies as one moves from the proximal to distal region of the basal body.

Each mature basal body is associated with a probasal body that will elongate during the next mitotic cycle [77], [78]. In wild-type cells, the probasal bodies are roughly 80 nm in length and consist of a proximal ring of amorphous material, a nine-spoked cartwheel and nine triplet microtubule blades [79]. In *bld2-5* cells, the proximal ends of the probasal bodies maintain a ring of amorphous material and a cartwheel, however microtubule blades may be incomplete (Figure 5; Movie S1). Unlike the probasal bodies, the amorphous material in the mature *bld2-5* basal body is not present as a thin ring rather it can extend to over 200 nm, which is variable from cell to cell. Most tomographic reconstructions have both amorphous material and microtubule blades (Figure 5A–E; arrowheads; Figure S3; arrowheads). The assembly of microtubule blades is also incomplete with singlet, doublet and sometimes triplet microtubules present as one moves from the proximal base of the basal body to the distal end (Figure 5; right; Figure S3). Incomplete basal bodies were also observed with only 7 or 8 blades are present at the distal tip (Figure S3A). In some cells the cartwheel structure assembles farther from the proximal base than in wild-type basal bodies (Figure 5 C, D; arrow; Figure S3C; arrow). Basal bodies competent to template flagella assemble ectopic transition zone material in the basal body proper (Figure S3B, D; arrow), which resembles the ectopic transition zone present in the *uni3-1* strain [47]. Probasal bodies in the *bld2-5* strain assemble with minor defects; however, as the basal body matures, it loses its integrity, which indicates that Bld2p is needed to maintain basal body structure. The collapsed centrin fibers and aberrant rootlet microtubules observed by immunofluorescence are confirmed by the tomography in which the fibers are misplaced in many of the cells.

### Taxol Supersensitive Strains Recover from Taxol Treatment

Mutant and wild-type cells were treated with 8 µM Taxol for 48 hours, washed twice in rich medium and stained with Trypan blue to determine the number of dead cells in each replicate sample (n = 3). There was no significant difference between the average number of live cells in treated and untreated cultures of *bld2-5*, *bld2-6*, *tub2-1*, *pf15-1* and *pf15-3* strains (Table 2). These data suggest that the Taxol supersensitivity of these mutants does not arise from a basal body integrity checkpoint but rather from an arrest.

**Table 2:** Average number of viable cells after 48 hr exposure to 8 µM Taxol.

Strain	Rich Medium	Rich Medium+Taxol	*p* value
*BLD2*	245±12.2	224±27	0.16
*bld2-5*	271±20.8	263±17.2	0.17
*bld2-6*	270±27	264±40.6	0.85
*tub2-1*	245±32.6	243±25.2	0.91
*pf15-1*	266±15.2	246±18.2	0.16
*pf15-3*	232±15.8	222±19.7	0.67

Cells were treated with 0.4% Trypan blue and examined by phase microscopy. 300 cells from three independent samples were counted. For each strain, the number of the viable cells in media (failing to stain with Trypan blue) with and without Taxol was compared by a Student’s *t*-test to establish a *p* value.

### Katanin is Mislocalized in the *bld2* Mutants

**Figure 6:**
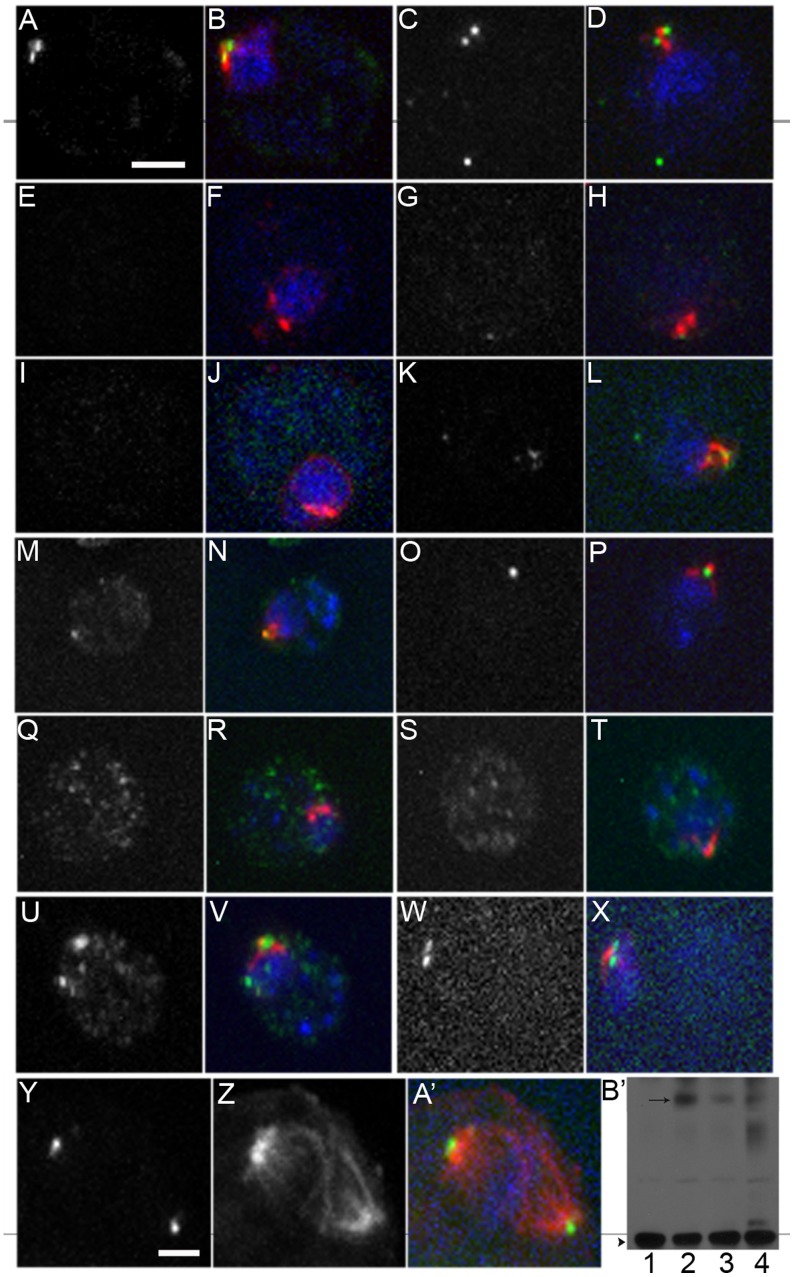
Katanin p80 is absent or diffusely localized in the *bld2* mutant strains. (A–D) Katanin p80 localizes as two dots (green) near centrin (red) in wild-type cells. DNA is stained with DAPI (blue). (E–H) *bld2-6* cells lack or show diffuse katanin p80 staining. (I–L) in the *bld2-5* strain has heterogeneous katanin p80 staining that is correlated with the centrin localization pattern. Katanin p80 localization appears wild-type in *bld2-7* (M, N) and *bld2-9* (O, P) pseudorevertants. *bld10* cells do not localize katanin p80 (Q, R), while *uni3* cells show variable staining (S–V). Katanin p80 localization appears wild-type in *uni1* cells (W, X). Katanin p80 localizes to the spindle poles during mitosis where the spindle microtubules (red) are stained with an antibody against α-tubulin and DNA (blue) is stained with DAPI (Y–A’). (B’) Immunoblot of katanin p80 (arrow) and PbsA (arrowhead) in lysates from wild-type (1), *pf15-1; PF15HA* (2), *bld2-5; PF15HA* (3), and *bld2-6; PF15HA* (4) cells. Cell lysates of wild-type and mutants show no difference in the level of katanin p80 as standardized by PbsA. Scale bar in Panel A equals 5 µm. Panels A–X are at the same magnification. Scale bar equals 2 µm. All images are at the same magnification.

Katanin influences microtubule dynamics by severing microtubules. The *PF15* gene encodes p80 katanin [56] and *pf15* strains show Taxol supersensitive (Figure 1A, B). We constructed a PF15-HA epitope tagged vector that places the HA tag at the terminal amino acid, which was changed from a leucine to an asparagine, transformed the plasmid into the *pf15-1* strain, and screened for rescue of the paralyzed flagellar phenotype. Twenty-one independent swimming strains were isolated and each transgenic strain also rescues the Taxol supersensitive phenotype. Immunoblots were used to determine which transgenic lines have the highest level of PF15-HA protein. Seven of the twenty-0ne strains showed a band detected by antibodies to HA antibody with the correct molecular weight (∼100 kDa). Based on immunoblot and immunofluorescence data, one of the lines was used for further analysis (Figure 6).

**Figure 7:**
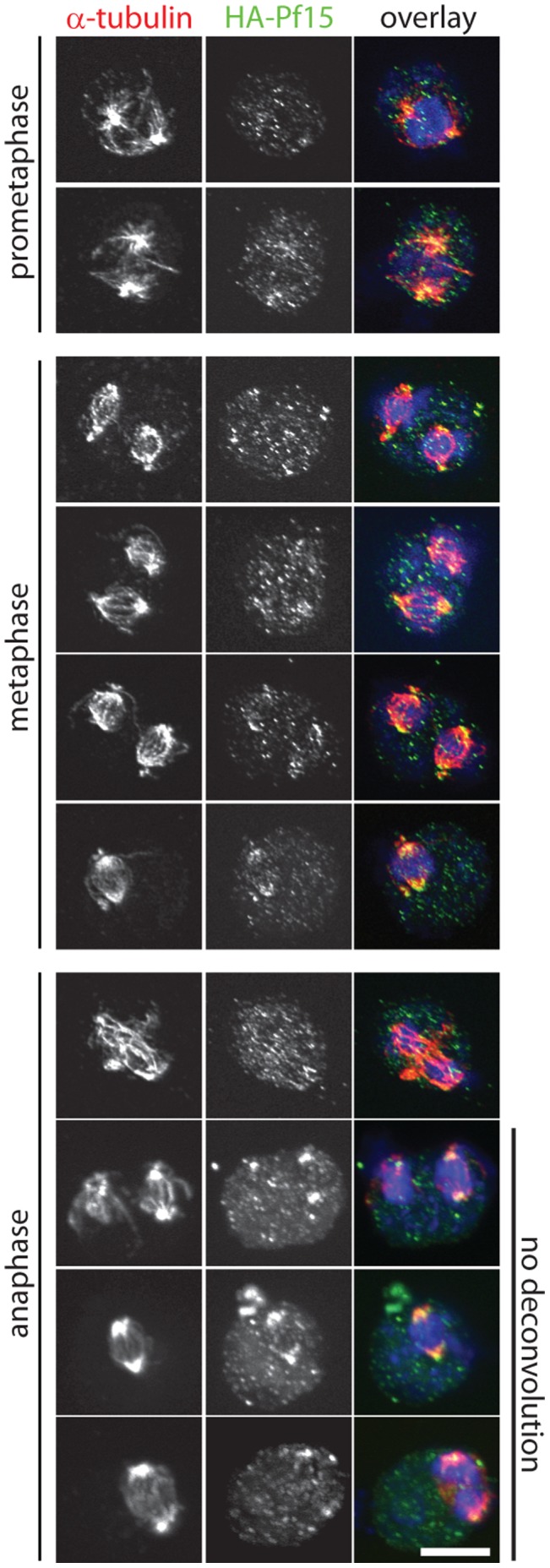
Katanin localizes to the spindle poles in mitotic cells. Synchronized cultures were fixed and stained for α-tubulin (red, left panel), HA (green, middle panel), and DNA (blue). Cell cycle stages were determined based on the DNA and tubulin staining patterns. The prometaphase, metaphase, and top row of anaphase are deconvoluted maximum projections of the z-stack. The bottom three rows for anaphase are maximum projections of the z-stack without deconvolution. Scale bar equals 5 µm.

By immunofluorescence, katanin localizes as two dots in interphase (n = 57, (Figure 6A–D) and to the spindle poles during mitosis (n = 4, Figure 6Y–A’; Figure 7) in wild-type cells. Sixty-six percent of *bld2-6* cells (n = 70) lack katanin p80 staining and the remainder have diffuse, lower intensity staining (Figure 6E–H). The *bld2-5* cells (n = 30) show two centrin localization patterns (Figure 2C, D), which are correlated to the katanin localization pattern. Of the cells with collapsed centrin, the majority (n = 12/14) has no katanin p80 localization while the remaining two have diffuse katanin p80 at the basal bodies (Figure 6I, J). Of the cells with wild-type centrin fibers (n = 16), 4 lack katanin p80 staining and 8 show diffuse katanin p80 localization (Figure 6K, L). The remaining 4 cells show wild-type katanin p80 localization. Intragenic revertants display wild-type katanin p80 localization (n = 40) (Figure 6P–S). Immunoblots show that the amount of katanin p80 protein in the *bld2* cells is similar to the expression in wild-type cells. Therefore, the lack of localization is not due to a decrease in katanin p80 expression (Figure 6B’). Intact basal bodies appear to be important for katanin p80 localization.

### Katanin Localization Requires Triplet Microtubules, but not the Transition Zone

To further determine which structures in the basal body apparatus are needed for katanin p80 localization, we examined p80 localization in *uni1*-2 cells, which lack a transition zone on the daughter basal body [26] in *uni3-1* cells, which assemble doublet but not triplet microtubules [22], [80]; and in *bld10* cells, which lack any microtubule blades [23]. Katanin p80 localization in *uni1-2* cells appears as two dots (n = 6), which suggests it does not require the transition zone for proper localization (Figure 6Q, R). In *bld10-1* cells, there is no localization of katanin p80 (n = 30), which further supports the need for the basal body for localization of katanin. Localization of katanin p80 in the *uni3* cells shows a variety of phenotypes that include the complete absence (40/50), diffuse staining (5/50) or increased signal (5/50) (Figure 6S–V). Because *uni3-1* cells lack triplet microtubule blades except at the distal tip of the basal body [22], it seems likely that triplet microtubules are important for katanin p80 localization but are not sufficient.

**Figure 8:**
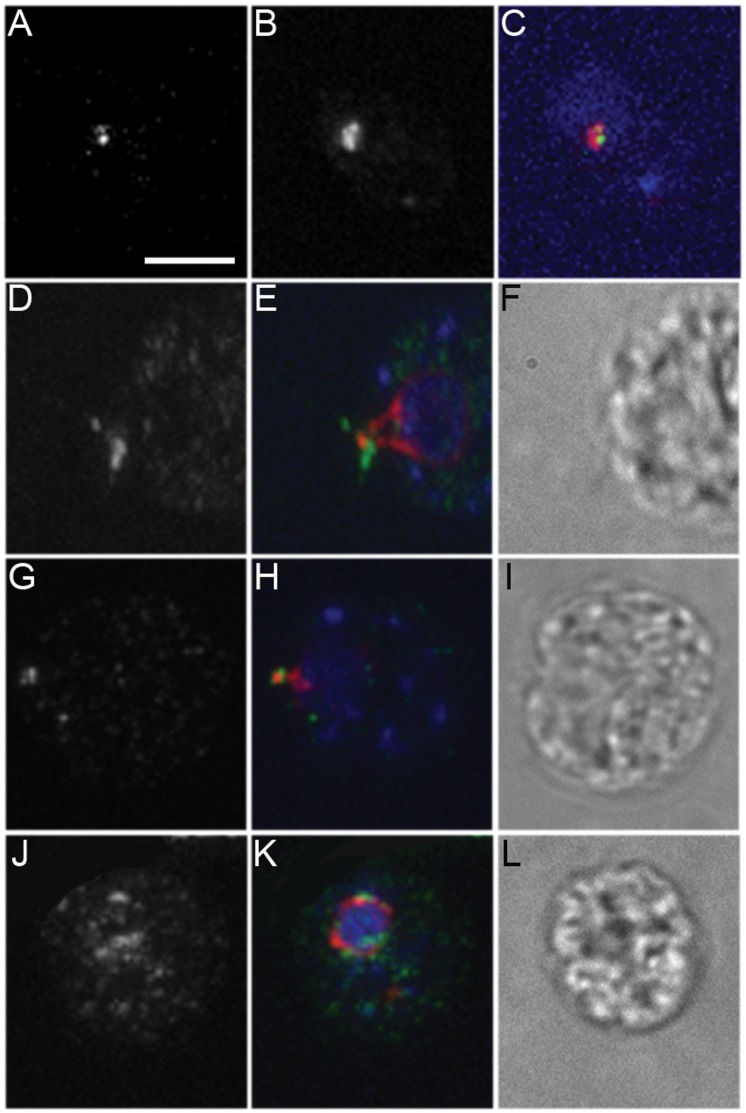
IFT74 localization is aberrant in the *bld2* alleles. (A–C) IFT74 and katanin p80 do not colocalize. (D–F) IFT74 (green) localizes to the base of the flagella in wild-type cell as a band as well as to the proximal region of the flagella and partially colocalizes with centrin (red) in the striated fiber at the distal end of the basal body but not along the nucleo basal body connectors at the proximal end (red). (G–I) *bld2-5* cells show staining at the base of the flagella, however the localization appears reduced compared to wild-type cells. (J–L) IFT74 localizes throughout the cytoplasm in *bld2-6* cells. In about one-half of the cells examined, IFT74 localizes near the aberrant centrin staining at the nucleus. DNA (blue) is stained with DAPI. Scale bar in Panel A equals 5 µm. Panels A–L are at the same magnification.

IFT52 localizes to transition fibers and localizes aberrantly in the *bld2-1* allele [20], we examined localization of another component of the IFT B complex, IFT74. As shown previously [61], IFT74 localizes to the flagellar base as a punctate dot and in the proximal region of the flagella in wild-type cells (Figure 8D–F). IFT74 and katanin p80 do not colocalize in wild-type cells (Figure 8A–C). This result supports the *uni1-2* result that katanin p80 is not present on the transition fibers.

Like the katanin p80 localization, the IFT74 pattern in *bld2-5* is more diffuse than in wild-type cells (n = 40, Figure 8G–I). In *bld2-6* cells, there is increased staining throughout the cytoplasm of all cells with dots that are not at the basal body region (n = 40) (Figure 8J–L). Sixteen cells show IFT74 localization near the basal bodies. The increased cytoplasmic staining in *bld2-6* cells may reflect an increased level of IFT74 in the cytoplasm that would normally be present in the flagella.

## Discussion

### Loss of Basal Body Integrity Perturbs Katanin Localization

The *pf15* and *pf19* alleles are unique among the flagellar motility mutants tested in *Chlamydomonas*; they confer supersensitivity to Taxol (Figure 1). The mutants with basal body integrity defects (*bld2*, *bld10*, *bld12, uni3*) or basal body fiber defects (*vfl1*, *vfl2*, *vfl3*) also confer Taxol supersensitivity and suggest a simple hypothesis that we tested. These organelles help to recruit proteins for spindle assembly and function. Specifically, we showed that a loss of basal body integrity results in a failure to recruit and localize katanin and this failure mimics the loss of function phenotype of the *pf15* and *pf19* strains. Acetylation, a post-translational modification of K40 in α-tubulin, is associated with more stable microtubules [81], and the Taxol supersensitivity phenotype of a β-tubulin mutant [2] or in the katanin mutants in *Tetrahymena* [7] is associated with increased levels of acetylated α-tubulin. The *bld2* alleles do not have increased acetylation of interphase microtubules (Figure 3).

We identified viable null alleles in the *PF15* and *BLD2* genes, which suggest that these two genes are not essential in *Chlamydomonas.* The *bld2-6; pf15* double mutant confers a more severe defect than in either single mutant on Taxol medium. This more severe phenotype indicates that recruitment of additional proteins at basal bodies affects microtubule dynamics. None of the mutants block spindle function in mitosis in the absence of Taxol. Given that the *bld2-1* and *bld2-6* alleles have a recessive meiotic defect, there may be a stronger requirement for the recruitment of proteins to the meiotic spindle than to the mitotic spindle in *Chlamydomonas*. Although mutants with basal body integrity defects can still build spindles and progress through the cell cycle, microtubules in these cells may be inherently unstable as judged by Taxol sensitivity.

Electron tomography of the *bld2-5* allele suggests that ε-tubulin is necessary for basal body assembly/elongation. This staggered phenotype of *bld2-5* is also observed in the *bld2-1; rgn1-1* strain, where *rgn1-1* is a partial suppressor of the *bld2-1* allele [34], and it is similar to the knockdown phenotype observed in *Paramecium* [82]. Since the probasal body structure is not affected to a large degree, this allele suggests that this mutant ε-tubulin is able to participate in the initiation events needed to build the probasal body, but not in the elongation of the probasal body to the daughter basal body. Based on tomographic reconstruction of duplicating basal bodies, these two events are separated in time. The existing probasal body elongates to become the daughter basal body at prophase and the new probasal body forms in metaphase (O’Toole and Dutcher, in preparation).

Rasi and colleagues reported that katanin p60 is an essential gene, is required for release of the basal bodies from the flagella using an RNA interference strategy to the p60 gene, and that katanin localizes to the basal bodies [83]. However, Dymek and Smith suggest that katanin p60 is encoded by the *PF19* gene [84]. Based on two *pf19* alleles, this gene does not play a role in release of flagella, and the anti-p60 serum reported by Rasi does not recognize the tagged p60-GFP protein in whole cell extracts [84], which makes our tagged gene the only means to examine localization of the katanin heterodimer. Our phenotypes for *pf15* and *pf19* are similar and suggest that katanin p60 and p80 behave similarly in *Chlamydomonas* as in other organisms.

### Localization of Katanin Requires Intact Basal Bodies

The transition fibers are required for IFT localization, and subdistal appendages are required for PCM localization in animal cells, several of our observations were unexpected. In *uni1-2* cells, which lack the transition zone and transition fibers on the younger of the two basal bodies, two dots of katanin are observed rather than one. Additionally, IFT74 and katanin p80 do not colocalize. Thus, we suggest that the transition fibers are not required for katanin p80 localization and that the requirements at the basal bodies for IFT and katanin p80 recruitment and localization are different.

We suggest that the recruitment requires triplet microtubules as the *uni3* mutant shows defects in p80 localization but has normal transition fibers. The *uni3* mutant fails to assemble triplet microtubules, but 25% of the cells assemble two flagella, 25% assemble one flagellum, and 50% have no flagella. We have hypothesized that the age of the basal bodies influences this distribution [22], [47]. As the basal bodies age, they may acquire additional proteins or post-translational modifications that allows for recruitment. Thus, the katanin localization phenotype is intriguing in that there are three localization phenotypes in *uni3* mutants. These may correspond to the age of the older basal body. The triplet microtubules are known to be important for the localization of centrin and rootlet microtubules via specific microtubule blades [47]. We cannot rule out the model that the triplet microtubules do not directly recruit p80, but may play an indirect role via intermediary proteins. Basal bodies in *C. elegans* and *Drosophila* lack triplet microtubules, but still recruit PCM. Like *Chlamydomonas*, basal body mutants in these organisms also fail to recruit PCM and suggest the mechanism of PCM recruitment may depend on the basal body structure of an organism.

### Other Proteins that Moderate Taxol Sensitivity

Besides basal body defects, work in other systems has shown multiple modes to confer Taxol sensitivity. Interestingly, two smoothened antagonists sensitize cells to Taxol in ovarian cancer cell lines [85]. It is interesting to consider that localization of the hedgehog pathway in the cilia could modulate recruitment of other proteins to the basal bodies. Increased expression of Nek4, a NimA-like kinase is associated with Taxol sensitivity [86]. Its targets remain unknown. Finally, changes in expression of Septin10 and Bub3 alter Taxol sensitivity. Increased Sept10 expression is associated with sensitivity while decreased expression is associated with resistance to Taxol [87], [88]. Modifiers that confer Taxol sensitivity have been identified in HapMap lymphoblastoid cell lines; the predominant class of genes with variants encodes solute carriers (SLC) [89]. Resistance to Taxol has been observed in a large number of clinical samples following treatment with this drug. Mutations in over 20 amino acids in β-tubulin have been observed in Taxol resistant cell lines [90]. There may be many targets and mechanisms by which Taxol sensitivity is modulated. Further screens for the Taxol supersensitivity phenotype may provide a new class of variants that will be useful for cancer therapeutics.

## Supporting Information

Figure S1 **Schematic drawing of the katanin p80 epitope-tagged transgene.** The last amino acid and the stop codon were mutated to a *Not*I restriction site by knitting PCR. The 3X hemmaglutinin (HA) tag was introduced into the engineered site.
(TIF)

Figure S2 **Rescue of the meiotic phenotype requires two wild-type copies of the** ***BLD2*** **gene and the** ***BLD2*** **transgene does not rescue the mitotic lethality.** Cross I is a repeat of the results obtained previously [34] showing that the disomic strain (red and black chromosomes) produces no viable progeny when crossed by wild-type strain (CC-124, blue) (n = 120 tetrads). Cross II involves a wild-type CC-1952 parent (green chromosome) carrying the *BLD2* transgene (purple) that is unlinked to the *BLD2* locus. The presence of the transgene is sufficient to rescue the meiotic phenotype (75% of the progeny from 40 tetrads survive). This result is reinforced by results in Cross III that uses progeny from Cross II that lack the *bld2-1* chromosome, which were eliminated from consideration using PCR and digestion with *Fok*I (Materials and Methods). This leaves six possible genotypes. No aflagellate progeny were recovered, which strongly suggests that the *bld2-4* allele is lethal (indicated by 0 under viable progeny for strains iiii and iiiiii). dCAPS markers described in Tables S1 and S2 were used to determined which strains carried CC-1952, *bld2-4* chromosomes, and the *BLD2* transgene. Ten strains were used for Cross III. Three of them had the CC-1952 chromosome with the transgene gave greater than 92% viable progeny in 25 tetrads. Two of them had the CC-1952 chromosome and no transgene and gave 89% viability in 25 tetrads. Three progeny had the *bld2-4* chromosome, the CC-1952 chromosome, and the transgene and gave 60% viability in 125 tetrads, but no aflagellate progeny were recovered, which suggests that the *bld2-4* allele is lethal. Two progeny had the *bld2-4* chromosome and the CC-1952 chromosome and gave 24% viability in 100 tetrads. Again no aflagellate progeny were recovered.
(TIF)

Figure S3 **Serial thin sections through** ***bld2-5*** **basal bodies show structural variation.** A–C. Serial, 80 nm sections of *bld2-5* basal bodies shown in cross section from three different cells (proximal-distal, left-right). The proximal basal body contains dark, amorphous material surrounding partial microtubule blades (A, arrowheads). The assembly of blades can be incomplete with singlet, doublet and triplet blades as one moves from the proximal to the distal tip. (C) The cartwheel is abnormally assembled in the middle of the basal body rather than the proximal base (arrow). (B,D) Some cells show ectopic transition zone material assembled in the basal body proper, shown in cross section (B, arrow) and longitudinal view (D, arrow). Scale bar equals 200 nm.
(TIF)

Table S1 **Primers used for mapping** ***bld2-4*** **to a 100 kb region and** ***bld2-5*** **to a 54.1 kb region of** ***Chlamydomonas reinhardtii*** **(JGI version 5.3).**
(DOCX)

Table S2 **Primers used to delineate the deletion in the** ***bld2-6*** **strain.**
(DOCX)

Movie S1 **A movie of serial, tomographic slices through the complete volume of** ***bld2-5*** **basal bodies.**
(MOV)
