# Correction: Katanin Localization Requires Triplet Microtubules in *Chlamydomonas reinhardtii*

**DOI:** 10.1371/journal.pone.0145569

**Published:** 2016-01-15

**Authors:** Jessica M. Esparza, Eileen O’Toole, Linya Li, Thomas H. Giddings Jr, Benjamin Kozak, Alison J. Albee, Susan K. Dutcher

The authors would like to correct Fig 1, as errors were introduced in the preparation of this figure for publication. In Fig 1B, the *bld2-6; BLD2* panel and the *pf15-1; PF15HA* panel are duplicates. The authors regrew the cell cultures in these panels, as well as the parental strains shown in the same panels of Fig 1A, and they have provided a corrected version of Fig 1 here.

**Fig 1:**
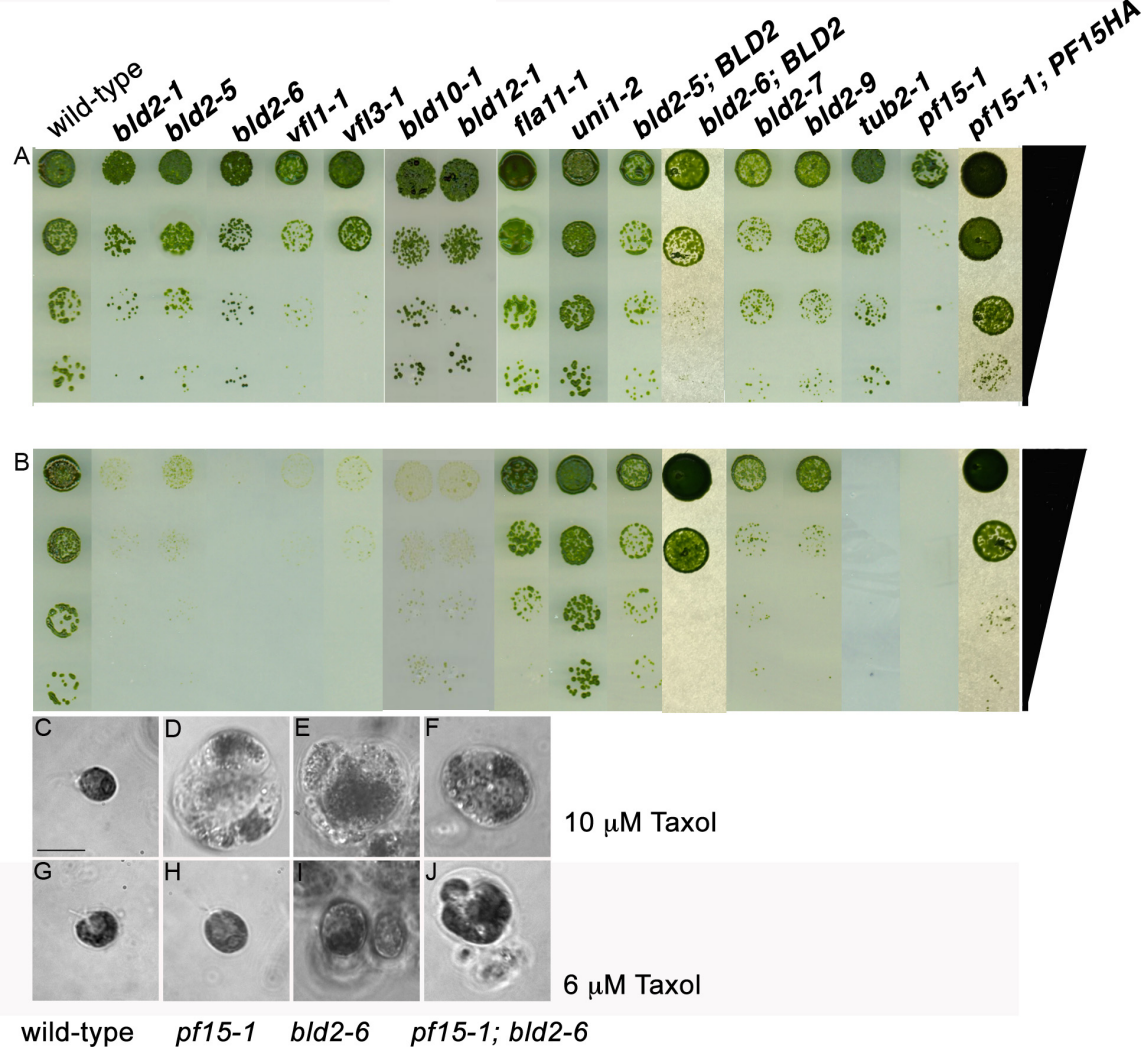
Basal body mutant strains show supersensitivity to Taxol. (A) Serial dilution of mutant, rescued, and intragenic revertant strains on control medium and (B) 8 μM Taxol-containing medium. Phase images of cells on media with different Taxol concentrations. (C, G) Wild-type, (D, H) *pf15-1*, (E, I) *bld2-6* and (F, J) *bld2-6*, *pf15-1* double mutant on 10 μM (C–F) or 6 μM Taxol (G–J) containing medium. The *bld2-6*, *pf15-1* double mutant is unable to grow on 6 μM Taxol containing medium compared to the single mutant strains. Scale bar in Panel C equals 10 μm. Panels C–J are at the same magnification.

The authors confirm that these changes do not alter their findings. The authors have provided the underlying images as Supporting Information.

## Supporting Information

S1 Fig Underlying data. Serial dilutions of mutant and rescued strains for *bld2-6; BLD2; bld2-6* and *pf15; PF15*::*HA* on control medium (A) and 8 μM Taxol-containing medium (B).
(TIF)
