# Supplementary figures and images for: Katanin Localization Requires Triplet Microtubules in Chlamydomonas reinhardtii
Source: PLoS One. 2013 Jan 8;8(1):e53940. doi: 10.1371/journal.pone.0053940 (PMC3540033; doi:10.1371/journal.pone.0053940)

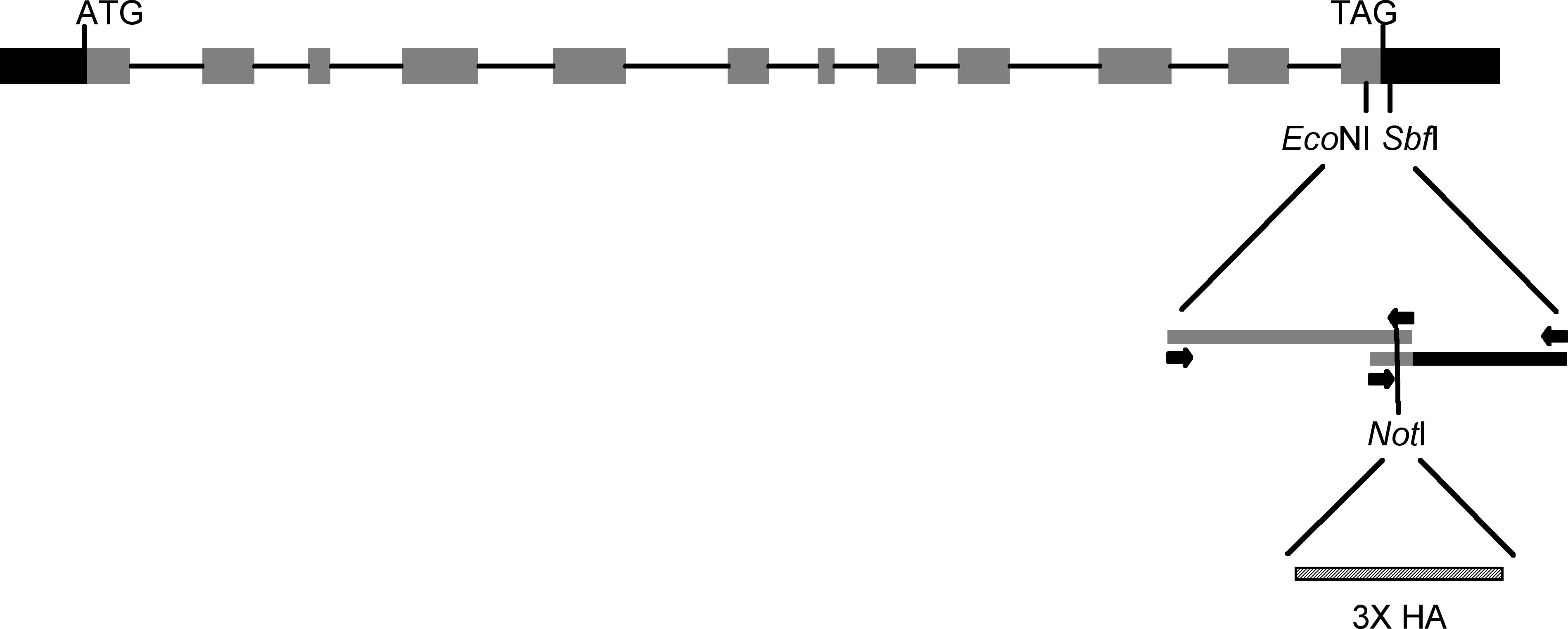

Supplement: Figure S1 — Schematic drawing of the katanin p80 epitope-tagged transgene. The last amino acid and the stop codon were mutated to a NotI restriction site by knitting PCR. The 3X hemmaglutinin (HA) tag was introduced into the engineered site. (TIF) [file pone.0053940.s001.tif]

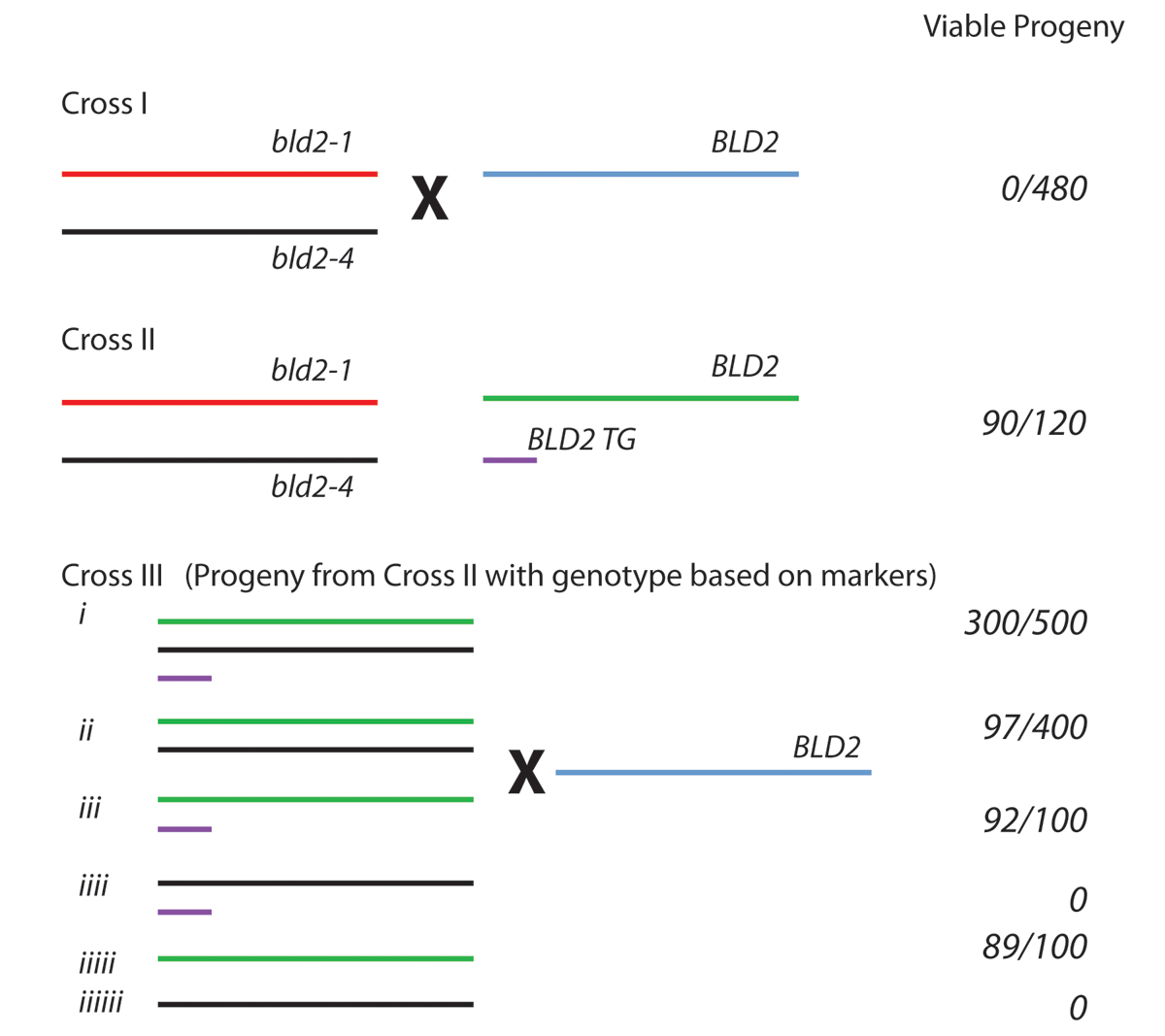

Supplement: Figure S2 — Rescue of the meiotic phenotype requires two wild-type copies of the BLD2 gene and the BLD2 transgene does not rescue the mitotic lethality. Cross I is a repeat of the results obtained previously [34] showing that the disomic strain (red and black chromosomes) produces no viable progeny when crossed by wild-type strain (CC-124, blue) (n = 120 tetrads). Cross II involves a wild-type CC-1952 parent (green chromosome) carrying the BLD2 transgene (purple) that is unlinked to the BLD2 locus. The presence of the transgene is sufficient to rescue the meiotic phenotype (75% of the progeny from 40 tetrads survive). This result is reinforced by results in Cross III that uses progeny from Cross II that lack the bld2-1 chromosome, which were eliminated from consideration using PCR and digestion with FokI (Materials and Methods). This leaves six possible genotypes. No aflagellate progeny were recovered, which strongly suggests that the bld2-4 allele is lethal (indicated by 0 under viable progeny for strains iiii and iiiiii). dCAPS markers described in Tables S1 and S2 were used to determined which strains carried CC-1952, bld2-4 chromosomes, and the BLD2 transgene. Ten strains were used for Cross III. Three of them had the CC-1952 chromosome with the transgene gave greater than 92% viable progeny in 25 tetrads. Two of them had the CC-1952 chromosome and no transgene and gave 89% viability in 25 tetrads. Three progeny had the bld2-4 chromosome, the CC-1952 chromosome, and the transgene and gave 60% viability in 125 tetrads, but no aflagellate progeny were recovered, which suggests that the bld2-4 allele is lethal. Two progeny had the bld2-4 chromosome and the CC-1952 chromosome and gave 24% viability in 100 tetrads. Again no aflagellate progeny were recovered. (TIF) [file pone.0053940.s002.tif]

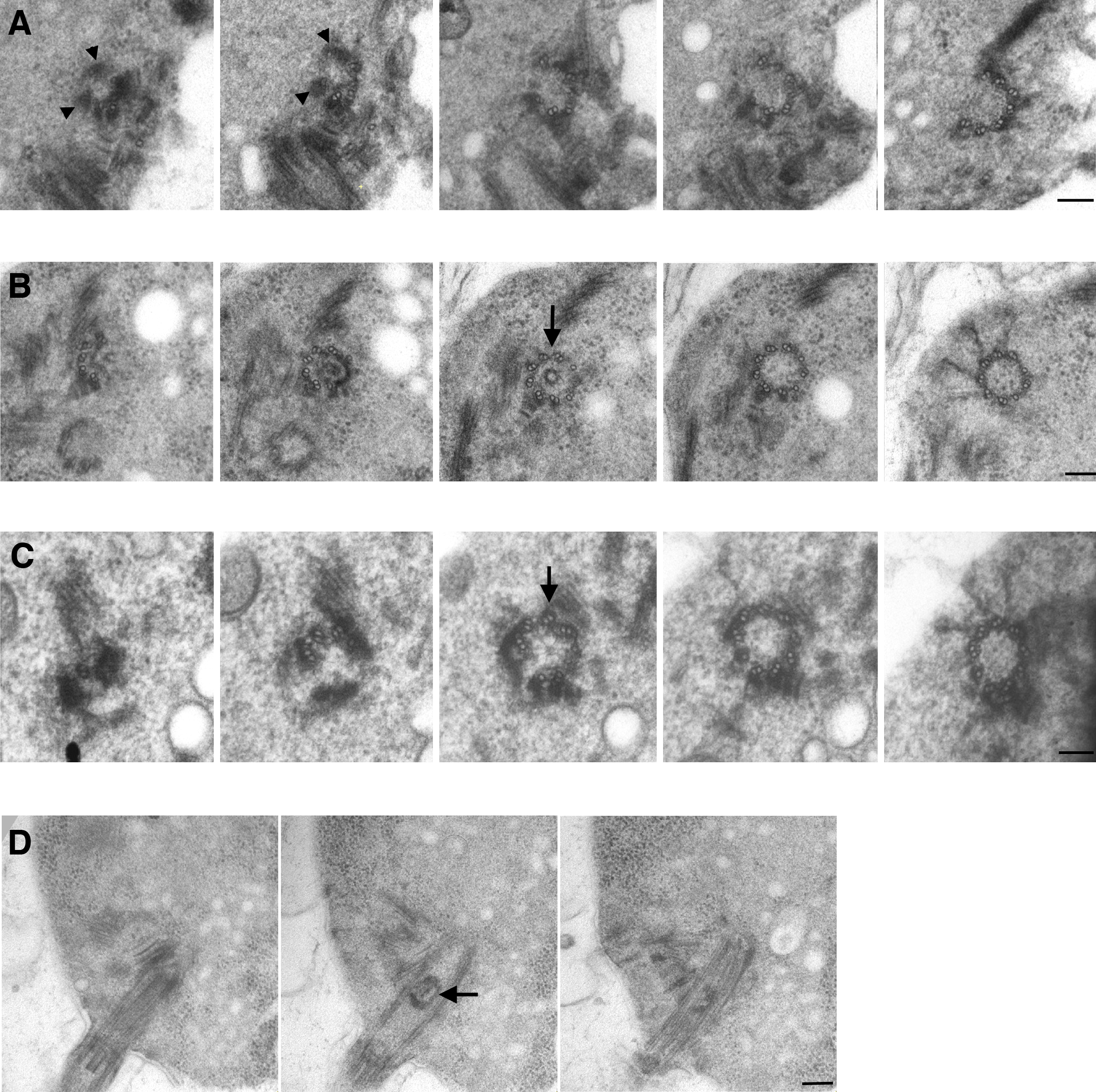

Supplement: Figure S3 — Serial thin sections through bld2-5 basal bodies show structural variation. A–C. Serial, 80 nm sections of bld2-5 basal bodies shown in cross section from three different cells (proximal-distal, left-right). The proximal basal body contains dark, amorphous material surrounding partial microtubule blades (A, arrowheads). The assembly of blades can be incomplete with singlet, doublet and triplet blades as one moves from the proximal to the distal tip. (C) The cartwheel is abnormally assembled in the middle of the basal body rather than the proximal base (arrow). (B,D) Some cells show ectopic transition zone material assembled in the basal body proper, shown in cross section (B, arrow) and longitudinal view (D, arrow). Scale bar equals 200 nm. (TIF) [file pone.0053940.s003.tif]
