# Supplementary material for: Katanin Localization Requires Triplet Microtubules in Chlamydomonas reinhardtii
Source: PLoS One. 2013 Jan 8;8(1):e53940. doi: 10.1371/journal.pone.0053940 (PMC3540033; doi:10.1371/journal.pone.0053940)
Supplement: Table S1 — Primers used for mapping bld2-4 to a 100 kb region and bld2-5 to a 54.1 kb region of Chlamydomonas reinhardtii (JGI version 5.3). (DOCX) [file pone.0053940.s004.docx]

**Supplemental Table 1.** Primers used for mapping *bld2-4* to a 100kb region and *bld2-5* to a 54.1kb region of *Chlamydomonas reinhardtii* (JGI version 5.3)

| **Name** | ***bld2-4*^a^** | ***bld2-5*^b^** | **Forward primer** | **Reverse primer** | **Enzyme/ Size** | **Position on CHR3**  **In (Mb)** |
| --- | --- | --- | --- | --- | --- | --- |
| NIT2 | C/B | 90/1548 | N/A | N/A | N/A | N/A |
| PGI | C/B | 52/85 | GCATCGCTCTCTCA  AAGCATGTAA | GTATTTTTGTGT  CGGTCCAGGGTC | Size | 4.379-4.380 |
| CYC-2 | C/B | 3/17 | GTGATCAGCCTGG  AGGAGTG | TCAAGCCAGCTC  AGTTGATG | *Hha*I | 4.271-  4.272 |
| 41.8-2 | C/B | 2/6 | TCGGGCGCTTAAA  CACTATT | TTCTCCTTGCCA  CTGAGGAT | *Scr*FI | 4.167 |
| 168:1281 | C | NT | AGGAGCAGCAGCA  GCCGCAGCTGCA | TACCCAGTGCTG  CTTCTTTCGCAT | *Pst*I | 5.362 |
| 46-172 | C | 0/12 | CTACCTCTTCAACGGGTTT  TGCTC | AACACTCTCACGCTTTTG  CGTAGG | Size | 4.105 |
| 46-165 | C | 2/23 | AGAGCACAGTTTGATCCG  GGTTCTA | CTATGTCTTGCAGCGCGT  TCTTG | *XbaI* | 4.098 |
| AMT5 | C | NT | TTCGTCGTAACCTCCACCAT | GTATACACAGCGCCACATGC | *Hha*I | 4.060 |
| GTP | C | 1/20 | CATCTTGGTCCTTGGCAAAC | GTTGTCGCGCCTACTTGC | Size | 4.038-4.039 |
| SEC7/  GEF2 | C | NT | AAAGAGTCATGGGGGAGGAG | GGTTCAGCACGTTCTGTGTG | *Msp*I | 4.026 |
| Sec7/  GEF3 | C/B | NT | GCAGATGTCGATGGAGCTAA | CCAGCACCTCCAACATGG | Size | 4.018-  4.019 |
| SEC61-1 | C/B | 1/3 | AAGCTTCTTTCCGGACTGCT | CCTTTCTGTTGCACTCACGA | *Msp*I | 3.912 |
| DMAT | C/B | 24/1456 | GGACATTCGTGTGGAGTGAA | GGGCACGTCTGACAGTAACA | Size | 3.734 |

^a^ For the *bld2-4*  disomic strain made with CC-1952, each marker was tested for heterozygosity (C/B) or hemizygosity (C) where C represents the polymorphic strain, CC-1952 and B represents the *bld2-4* allele originally induced in CC-125.

^b^ For the *bld2-5* allele, the number of recombinants progeny from a meiotic cross with CC-1952 is given over the total number of progeny scored for each marker.

^c^ Linkage group III in the Joint Genome Institute (JGI) version 5.3 of the Chlamydomonas Genome from original mapping to scaffold 9 in version 3.

NT=not tested.
