# Supplementary material for: Katanin Localization Requires Triplet Microtubules in Chlamydomonas reinhardtii
Source: PLoS One. 2013 Jan 8;8(1):e53940. doi: 10.1371/journal.pone.0053940 (PMC3540033; doi:10.1371/journal.pone.0053940)
Supplement: Table S2 — Primers used to delineate the deletion in the bld2-6 strain. (DOCX) [file pone.0053940.s005.docx]

**Supplemental Table S2.** Primers used to delineate the deletion in the *bld2-6* strain

| **Name** | **Forward primer** | **Reverse primer** | **Presence in *bld2-6*** |
| --- | --- | --- | --- |
| Cr058 1 | ACGATATCGCCATGGAAAAC | CCAACAGTCGCTCCCATACT | Yes |
| Cr058 4 | CACCTCGCTGCTATCTCCT | CCTGCCTCTGCTACTGCTG | Yes |
| Cr058 6 | GAGTCAGCCAAGACCAGGAG | TTGACAGCGCTGATGAACTC | Yes |
| Epsilon 20 | GCGGGTCACAAAGGAACATA | GTTTCGTGTGAGGCTCCTTC | No |
| Epsilon 21 | TGATGCAGCTCAGTCGAGAG | ACCTGCTATGCCTTGCTACG | No |
| Epsilon 22 | CAACGTAGCAAGGCAAGCA | TCTCATTGATGACGCCACAC | No |
| Epsilon 40 | TCACACGAAACACCAGCCTA | TCGCTCACCTTGAGTGTACG | No |
| Epsilon 41 | CGTACACTCAAGGTGAGCGA | CTCACGTCGCTCACCAACTG | No |
| Epsilon 26 | CATGCATGCTGAGACACACA | TGATTGCCATGACCTCTACG | No |
| Epsilon 27 | GCGTGTGTATGGCTGTATGC | GGTTCATGGTGATGTCGTTG | No |
| Epsilon 28 | GACTTTGCAATGCTCCCAGT | GGGTGTCAGGGGCTTACATT | No |
| Epsilon 29S/ Exon9R | TTCATGACGCTCTGATCCTG | ACCTGCGCTTGTACAGCTTG | No |
| Epsilon 31S/ Epsilon 34R | ATCGCACATACCTTCACGA | GCTCGTAGTGGTGCGTGTAG | No |
| Epsilon 45F/ Epsilon 32R | AATGTAAGCCCCTGACACCC | CAAGCGGTTCTCGTTCCTTA | No |
| PRMT 1 | GCCACACGTCTGTTTAGCA | TCCTTAAGCCCTTCTGATGC | No |
| PRMT 2 | CGCCATGCTCATGTTTACT | ACCAATGGTTCCCGGTCT | No |
| PRMT 3 | ACCAATACCAACACCCCAAA | GTATTGGCGCCGTTTCTAGT | No |
| PRMT 4 | GGTGGCTTGACACTGGAG | CCGGGCAACACCTAGGAC | N o |
| PRMT 5 | TTCGTCGTAACCTCCACCAT | GTATACACACGCCCACATGC | No |
| PRMT6 | TGCGAGAGTGGAGAGTGAAA | GTATGCGCACACGCATAGTC | No |
| PRMT 7 | CTGCAACAAGCACACACTCA | ATAGGCGTTGTTGAGGATGG | Yes |
| Primers for Tail PCR for cloning the breakpoint | |  |  |
| Ble 8 | TACGCCGAGTGGTCGGAGGT |  |  |
| Ble 15 | AGTGGTCGGAGGTCGTGTCC |  |  |
| Ble 16 | GTGGTCGGAGGTCGTGTCCA |  |  |
| Ble 25 | GGTCGTGTCCACGAACTTCC |  |  |
| Ble 26 | CCGAGGAGCAGGTACCCAAG |  |  |
| Ble 396 | CTGGACCGCGCTGATGAACA |  |  |
| Ble 389 | GCGCTGATGAACAGGGTCAC |  |  |
| Ble 349 | CGAAGTCGTCCTCCACGAAG |  |  |
| Ble 314 | AGCCGGTCGGTCCAGAACT |  |  |
