# Supplementary figures and images for: Correction: Katanin Localization Requires Triplet Microtubules in Chlamydomonas reinhardtii
Source: PLoS One. 2016 Jan 15;11(1):e0145569. doi: 10.1371/journal.pone.0145569 (PMC4714803; doi:10.1371/journal.pone.0145569)

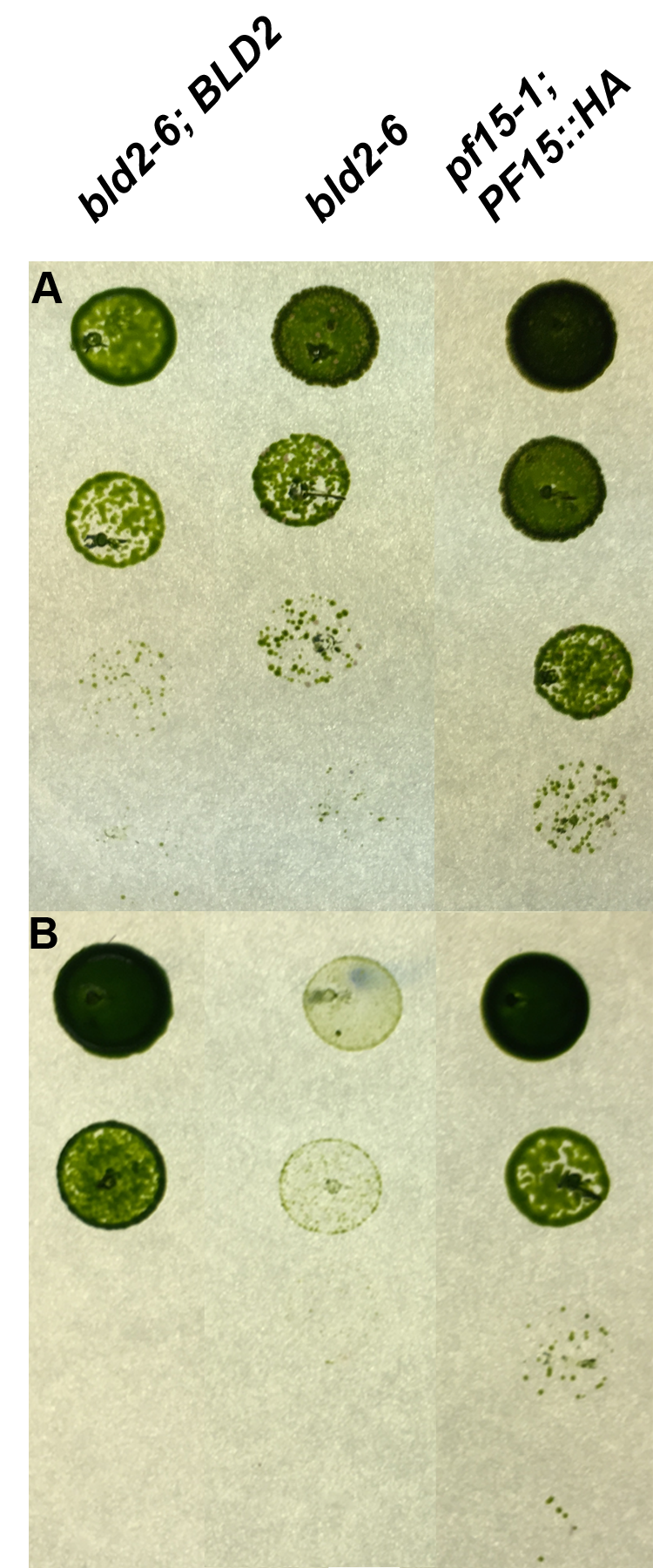

Supplement: S1 Fig — Serial dilutions of mutant and rescued strains for bld2-6; BLD2; bld2-6 and pf15; PF15::HA on control medium (A) and 8 μM Taxol-containing medium (B). (TIF) [file pone.0145569.s001.tif]
